# Recent advances on bonding mechanism in cold spray process: A review of single-particle impact methods

**DOI:** 10.1557/s43578-022-00764-2

**Published:** 2022-10-18

**Authors:** Moses A. Adaan-Nyiak, Ahmed A. Tiamiyu

**Affiliations:** Department of Mechanical and Manufacturing Engineering, University of Calgary, 2500 University Drive NW, Calgary, AB T2N 1N4 Canada

**Keywords:** Additive manufacturing, Adhesion, Spray deposition, Bonding, Coating, Powder processing

## Abstract

Cold spray (CS) processing is a layer-by-layer solid-state deposition process in which particles at a temperature below their melting point are launched to sufficiently high velocities to adhere to a substrate (and previously deposited particles), forming coatings/parts. Despite being in existence for over four decades, particle bonding mechanisms in the CS process are unclear due to the complex particle–particle/carrier gas interactions that obscure assessment. This review evaluates recent findings from single-particle impact approaches that circumvent these complexities and further provide new insights on bonding mechanisms. Theories on the evolution of oxide layer breakup and delamination, adiabatic shear instability, jetting, melting, and interface solid-state amorphization that contributes to bonding are assessed and carefully reviewed. Although there is a unified condition in which bonding sets on, this study shows that no singular theory explains bonding mechanism. Rather, dominant mechanism is a function of the prevailing barriers unique to each impact scenario.

**Graphical abstract:**

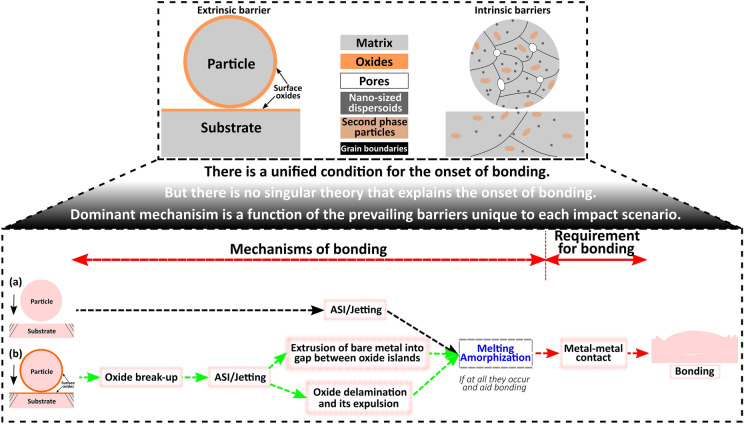

## Introduction and overview

Cold spray (CS) process is a solid-state deposition process in which microparticles are accelerated to supersonic velocities by pressured gas; these particles adhere to an oppositely positioned substrate and/or previously deposited particles to develop coatings/parts [1, 2]. CS is a member of the thermal coating techniques such as the high-velocity oxygen-fuel, flame spraying, and plasma spraying. However, microparticles during CS processing are launched at temperatures well below their melting point so that bonding relies on the kinetic energy of the particles rather than thermal, as is the case in thermal spray processing [3]. The no-melting particle requirement prior to impact in CS suppresses thermally induced defects such as stress, cracking of substrate and particle, oxidation etc [4, 5].

**Figure 1:**
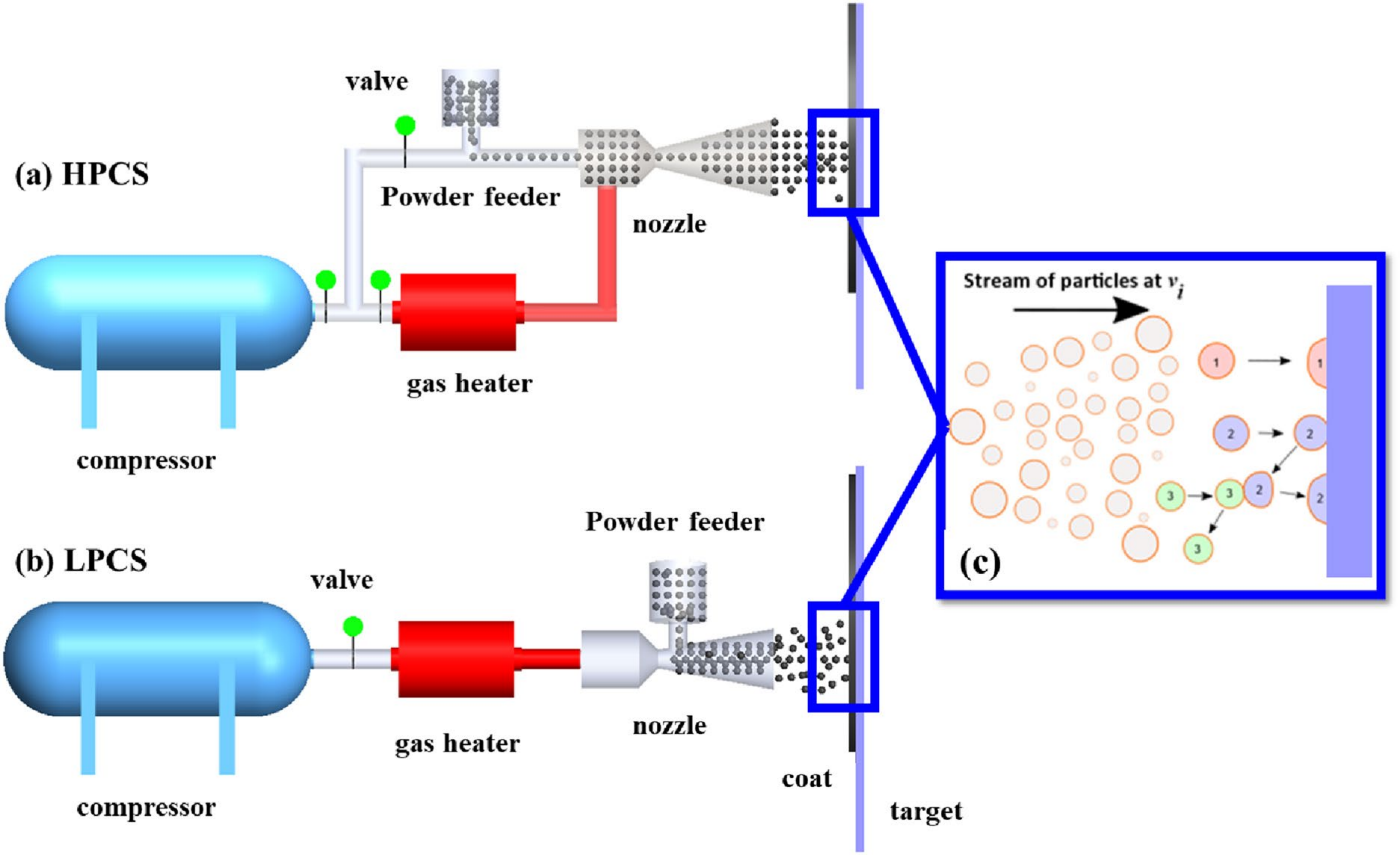
Schematic diagrams of (a) HPCS and (b) LPCS systems. (c) Schematic diagram of possible complex interactions that can occur in cold spray: during post-mortem analysis of CSed particles, it is difficult to distinguish the particles that arrive at the substrate along a straight trajectory (particle *1*) from those that are deposited ahead or behind due to possible particle–particle or particle–substrate-particle interactions (particles *1* vs *2*).

Based on the propulsive gas pressure requirement, CS is classified into two main types; the high-pressure cold spray (HPCS) and the low-pressure cold spray (LPCS) systems. In the HPCS system [Fig. 1(a)], the compressed gas whose pressure is greater than 1 MPa performs two distinct roles: (i) a portion of the gas transports the powder particles at the feeder to the de Laval nozzle and (ii) the other portion is heated in a chamber to boost the particle velocity. The gas-particle mixture and the heated gas are blended just prior to being injected into the nozzle throat beyond which the mixture expands through the long diverging section of the nozzle to generate the supersonic gas-particle stream.

Meanwhile, the compressed gas in an LPCS system that is heated before entering the de Laval nozzle has a typical pressure of less than 1 MPa [6] [Fig. 1(b)]. The powder feeder is situated at the diverging section of the nozzle. The heated gas mixes with the powder at the diverging section of the nozzle to form a particle-gas stream at low pressure. HPCS launches particles up to 800–1400 m/s, while LPCS are limited to 300–600 m/s particle velocities and can only be used to deposit lighter materials [7]. As the CS technology advances, other variants exist and have been reported in the literature; they include kinetic metallization, pulsed-gas dynamic spraying, and vacuum cold spray [8]. Readers are referred to the references for details about the variants.

### Processing parameters in cold spray

Like any manufacturing process, optimization of the CS process is necessary to improve process quality [9]. Although the material type and its constituting microstructural features contribute to the quality of deposits/particle interfacial bonds and the deposition rate, the processing parameters also strongly affect coat quality. These parameters are therefore categorized as follows:

1. *Propulsive gas parameters* (type of gas, gas temperature, and gas pressure): As stated earlier, the compressed gas in the CS process performs two key roles, to convey the powder particles, and to act as a propelling gas for the gas-powder mixture. The most widely used gases in CS include air, helium (He), and nitrogen (N_2_) [10]. In principle, He which is lighter in comparison with N_2_ is preferred in the CS process; this is because He achieves higher particle velocity required for bonding. However, He is expensive and it is not economically viable [11]; this results in the use of He and N_2_ mixture in some industrial applications. However, the snag of this approach is that N_2_ being diatomic is heavier, contributing significantly to the atomic mass of the gas mixture. The recommended ranges of gas temperature and pressure are 25–1000 °C and 0.5–6 MPa, respectively [6].
2. *Powder feeder parameters* (feed rate): Feed rate is the amount of powder metered into the spray nozzle per unit time [12]. The feed rate determines the nature of the gas-particle stream exiting the nozzle, and it has a direct influence on the particle impact velocity, ${v}_{\text{i}}$. The higher the powder feed rate, the lower the particle velocity due to severe gas–particle interaction [13], akin to those described in Fig. 1(c). Also, a higher feed rate results in a thicker and sharper profile of a single-track deposit. Powder feed rate between 10 and 30 g/s should be carefully selected for improved CS deposit quality [13, 14].
3. *Nozzle parameters*: The most important nozzle parameters include nozzle transverse speed, stand-off-distance (SoD)—the gap between the spray nozzle and the substrate, and spray angle—the angle between the nozzle central axis and the substrate [2]. The *nozzle transverse speed* affects the duration and quantity of powder impinging the substrate per unit time. Broadly speaking, lower nozzle transverse speed results in thicker coatings and sharper profile of a single pass deposit [15–18]. Also, low nozzle transverse speed increases the density, adhesion strength, and hardness of the deposit [19–21]. High nozzle transverse speeds are recommended in CS process because low nozzle transverse velocities contribute to high residual stresses at the deposit–substrate interface due to shot peening effect [6]. The thickness of a single pass deposit can be carefully controlled if the nozzle transverse speed and the feed rate are synchronously set [14]. Meanwhile, the ${v}_{\text{i}}$ and deposition efficiency (*DE*) increases with *SoD* and reduces after a critical optimal value; this also depends on the type of powder, for instance, 60 mm for Al and Ti and 110 mm for Cu [15]. A spray angle between 70 and 90° typically provides the highest *DE*, although 90° is the optimum. As the spray angle departs from the optimum, the normal velocity component contributes to the adhesion of the particle, while other velocity components contribute to the removal of the splats [22, 23]; this causes a decline in *DE* till it turns zero at the highest departure from the optimum [24].
4. *Feedstock material parameters* (particle -size, -distribution, -composition, -geometry, and -temperature): Decreasing the particle size increases the critical adhesion velocity, ${v}_{\text{cr}}$ [27]; this is postulated to be due to the high surface area-to-volume ratio in smaller particles that results in (i) lower kinetic energy [28], (ii) quicker heat conduction away from the bonding interface [7], and (iii) higher amount of adsorbents/oxides that hinder bonding [29]. Also, an increase in powder oxygen content impedes bonding and decreases the ductility of the deposit [10]. Metals such as aluminium, copper, zinc, silver, bismuth, and their alloys are commonly used in CS process because of their relatively low melting points and their ability to deform easily. The most controllable feedstock parameter is the *particle size*; it can be easily obtained by sifting through a sieve. However, *particle geometry* and topology are the hardest to control properties of the particle. The particle can be spherical, nearly spherical, sponge, dendritic, etc. [30]. The ${v}_{\text{cr}}$ of spray material would decrease if the *particle temperature* is increased, making bonding possible at relatively low impact velocities [31].

The summary of the processing parameters and their effects on the cold spray coatings properties are presented in Table 1.

**TABLE 1:** Influence of processing parameters on the cold spray deposit summary [1].

Parameter	Degree	Deposit strength	Adhesion	Deposit efficiency	Porosity	Residual stress
Gas pressure	↑	↑	↑	↑	↓	↑
Gas temperature	↑	↑	↑	↑	↓	↑
Gas molecular weight	↑	↓	↓	↓	↑	↓
Particle velocity*	↑	↑	↑	↑	↑	↓
Powder feed rate	↑	↓	↓	↓	↑	↑
Stand-off-distance	↑	**□**	**□**	**□**	**□**	**□**
Spray angle	↑	↑	↑	↑	↑	↑

*Although an increase in ${v}_{\text{i}}$ increases adhesion and DE, new findings show they decrease at very high velocity [25, 26].

↓ = Decrease ↑ = Increase □** = **No common view.

### Current trend, merits, and existing limitations of cold spray

**Figure 2:**
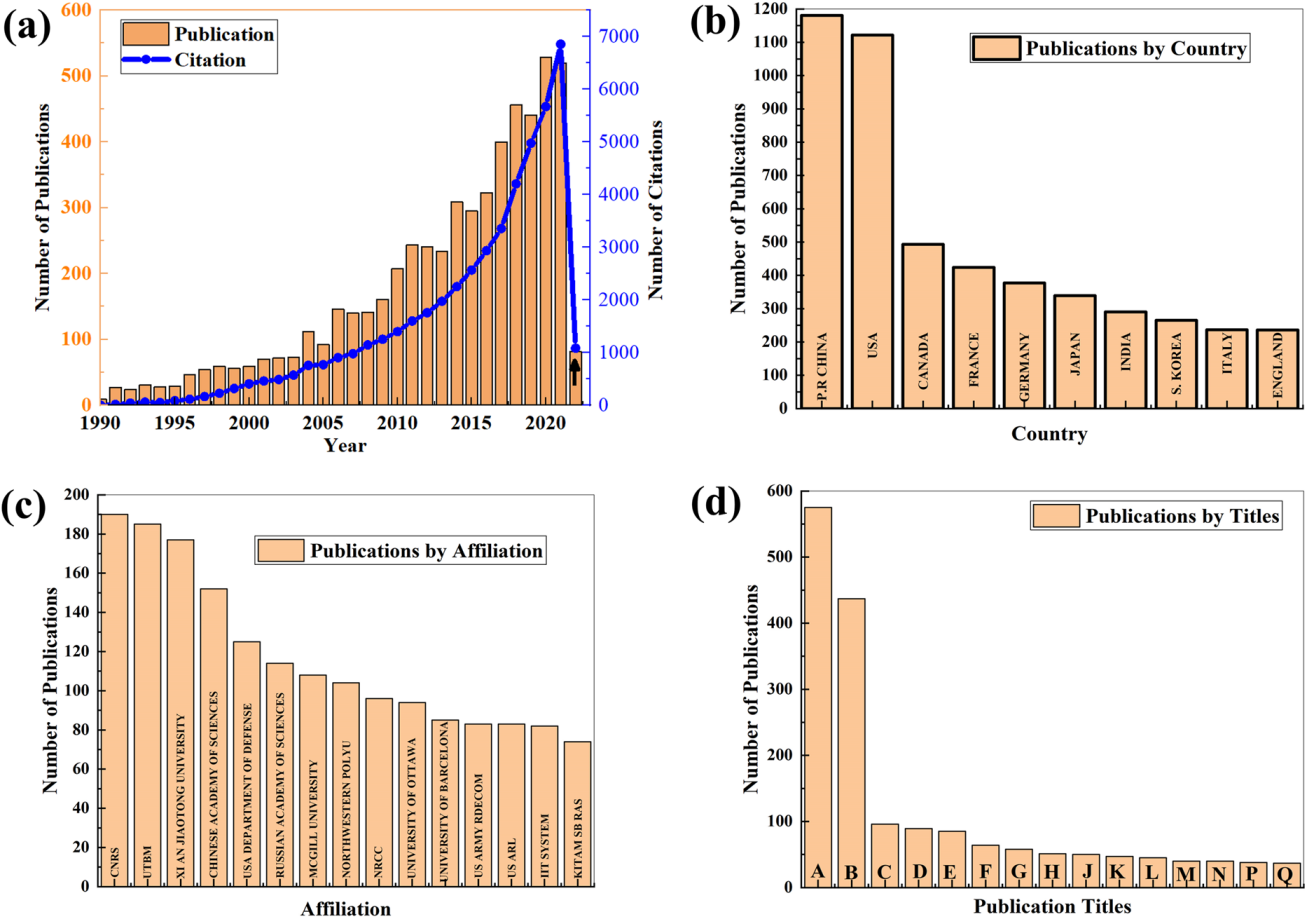
Research publications (a) research publication and citations per year, (b) research publication by country, (c) research publication by affiliation, and (d) research publication by publication titles. (*CNRS* Centre National De La Recherche Scientifique, *UTBM* Universite De Technologie De Belfort Montbeliard, *NRCC* National Research Council Canada, *RDECOM* Research Development Engineering Command, *ARL* Army Research Laboratory, *IIT* Indian Institute of Technology, *KITAM* Khristianovich Institute of Theoretical Applied Mechanics). (*A* Journal of Thermal Spray Technology, *B* Surface Coatings Technology, *C* Acta Horticulturae, *D* Applied Surface Science, *E* Surface Engineering, *F* Coatings, *G* Materials Science and Engineering a Structural Materials Properties Microstructure and Processing, *H* Materials Science Forum, *J* Journal of Alloys and Compounds, *K* Aip Conference Proceedings, *L* Materials Design, *M* Advanced Materials Processes, *N* Advanced Materials Research, *P* Acta Materialia, *Q* Fuel).

It’s been about four decades since the accidental discovery of this surface coating technology at the Institute of Theoretical and Applied Mechanics, Russian Academy of Sciences, Russia [29]. The vast benefits and potentials of CS have ignited the interest of scholars and those in the manufacturing sector. This has led to an exponential growth in research articles, reviews [32–37], patents and citations on the CS technology; many of the reviews focussed on the basic principles and applications of CS [7, 38]. These interests can be discerned from the Web of Science database which shows a near-steady rise in the number of publications and citations per year, beginning from the year 1990 [Fig. 2(a)]. Also shown in Fig. 2(b and c) are the top 14 countries and affiliations, respectively, where CS research is gaining interest and support. People’s Republic of China, USA, and Canada lead with 1181, 1122, and 493 publications, respectively. Meanwhile, Centre National De La Recherche Scientifique, Universite De Technologie De Belfort Montbeliard, and Xi’an Jiaotong University are the leading cold spray research affiliations with 190, 185, and 177 publications to their names, respectively. Also, most papers on CS technology are published in the Journal of Thermal Spray Technology (575), Surface Coatings Technology (437), Acta Horticulturae (96), Applied Surface Science (89), and other journals that suggest the expansion of CS to unconventional areas [Fig. 1(d)].

The potential of CS technology has ignited the interest of scholars due to its unique applications for corrosion coatings, in situ repairs of cracks, restoration/remanufacturing of unserviceable engineering/aeronautical components, etc. CS now finds extensive application in aerospace, energy, military, and biomedical, just to mention a few [38]. Interests in CS technology have also grown to be a potentially greener alternative as environmental and health & safety regulations become more stringent. It is also anticipated that the applications of CS technology will continue to expand to more applications that are non-traditional. These applications include photovoltaic (fabrication of complex conductive patterns in solar cells), wind power generation (enhancement of surface performance in components made of advanced polymer-matrix composites), medical (to apply bio-compatible material, such as hydroxyapatite [HAP], to substrates without compromising the integrity of HAP), and architectural (to create aesthetic metallic designs on any metal or ceramic substrate) [39].

Even though CS is young in the thermal coating family, the process has merits that distinguish it from other solid-state techniques [40, 41], including the ability to (i) support a variety of ductile metals [42], (ii) provide high-density, high-hardness, cold-worked microstructure [10], (iii) retain properties of initial particle materials [43], (iv) avoid oxidation and undesirable phases [10], (v) work with highly dissimilar materials [44], (vi) achieve high deposition efficiency as compared to other thermal coating processes [45], (vii) spray thermally sensitive materials [30, 43], and (viii) operate with high flexibility and precision control [40].

Like any manufacturing process, CS technology has its limitations, some of which include

1. The requirement for highly deformed particles during impact to aid bonding and subsequently, the development of coatings. This usually results in loss of ductility of the cold sprayed deposits [46], mandating a post-heat treatment in most cases.
2. Difficulty in processing intricate parts (especially internal features); CS is somewhat a “line-of-site” process [47].
3. Difficulty in processing harder materials; this is because CS is an all-solid-state deposition process which requires materials to be sufficiently ductile [48].
4. High rate of processing gas consumption [30]; this increases the operational cost of the system especially when expensive He gas is used.
5. Varying deposition rates resulting from nozzle obturation during prolonged spraying processes; this reduces coating quality [43].
6. Difficulty in coating/processing substrates with the large surface area due to small spray path/trace and short stand-off-distance [2].

### Mechanisms of bonding and objective of the study

In cold spray, the particle kinetic energy just before impingement is one of the key parameters in determining the quality of particle bonding and consolidation. It has been acknowledged that for a given material type and properties, there exists a critical velocity, ${v}_{\text{cr}}$, at or above which bonding is achieved [49]. At an impact velocity below the ${v}_{\text{cr}}$, the particle rebounds [25]. There are quite a few mechanisms of bonding proposed in the literature [29, 50] some of which are under debate. Some of these debates include whether or not adiabatic shear instability is required for bonding [51, 52], the roles of jetting—outward ejection of material from the particle–substrate interface, native surface oxide layer [29, 53], interfacial melting [54–56], and interface solid-state amorphization [57, 58]; these mechanisms will be examined in details in the latter section.

If at all CS technology will be extended to the uncharted applications listed above, the mechanism of bonding must be unambiguously clarified. In CS process, several microparticles are launched, and their complex interactions with carrier gas and other particles make it difficult, if not impossible, to measure the exact size and velocity of individual particles; this, in turn, makes the assessment of bonding mechanism difficult. An example of a typical complex particle interaction scenario is depicted in Fig. 1(c): *Particle 1* moving along a straight trajectory can impact and adhere to a substrate; *Particle 2* can hit the substrate without bonding but with reduced activation energy required for bonding upon rebound. As *Particle 2* rebounds, it can be hit back towards the substrate by an on-coming *Particle 3*, causing the adhesion of *Particle 2* to the substrate. It is therefore difficult to distinguish which of the particles arrive at the substrate along a straight trajectory or not in such possible events depicted in Fig. 1(c). Also, microparticle impacts occur at a very short time scale in the order of 10^−9^ s, so that impact-induced physical phenomena and microstructural evolutions that set on are obscured and are rather indirectly inferred from post-mortem characterization. Therefore, we theorize that the debates on the mechanism of bonding are likely connected to the lack of in situ techniques that can resolve bonding moments through real-time observations of single microparticle impacts.

To provide an in-depth understanding of this complex process, both numerical simulation and site-specific in situ experiments that isolate single macro/microparticle impact have been developed and conducted in the past few years. There are recent advancements such as access to new computational tools, a recently developed Laser-Induced Particle Impact Tester (LIPIT) that allows the launch of single microparticles and real-time observation of impact moments for well-known particle size and ${v}_{\text{i}}$, and the state-of-the-art characterization techniques that provide details of interfacial atomic distribution. These advancements have, in the last 5 to 10 years, provided new evidence on the mechanism of bonding that was not previously appreciated. Therefore, this paper presents an overview of recent efforts in understanding bonding mechanisms from the materials standpoint in single macro/microparticle impact experiments or simulations. To harness the full potential of CS technology for more industrial applications, it is hoped that the review will provide the CS community and those of other high-velocity impact processes (e.g. explosive welding, shaped charges), scholars, engineers, and managers both experience and inexperience, new insights on bonding mechanisms.

## Single-particle impact approaches

CS has evolved from just being a laboratory discovery to an industrial manufacturing process over time. The fact that the CS process involves the deposition of multi-particles at supersonic speeds and at a very short contact time makes in situ observations of single microparticle impacts challenging. There are now a couple of experimental approaches developed to understand the fundamental principles governing the physical phenomena that set on during CS processing [59–61]. Furthermore, the development of high-performance computing devices has caused growth in numerical efforts to understand particle–particle, particle–substrate, particle-gas interactions, as well as particle deformation, jetting, etc., that promote bonding in CS. The numerical investigation of microparticle impact and the associated extremely large strains and strain rates, and bonding provides a better perspective of this complex impact process that spans just a few nanoseconds. In this section, we briefly highlight the various numerical and experimental methods used to understand the unit process of CS to date.

### Numerical modelling and other computational methods

#### The Lagrangian method description

One of the first numerical approaches used in CS is the Lagrangian concept [62, 63]. A major advantage of the Lagrangian model is the ability to reduce the computation time on the assumption that the impact is symmetrical, hence allowing the use of axisymmetric (quarter) models [33]. In the Lagrangian model simulation, the projectile or particle mesh and the mesh at the impact region are made compact to obtain accurate results. Also, the movement of the mesh nodes together with the material enables precise tracking of the particle–particle and particle–substrate interfaces during impact. However, this model faces the challenge of high mesh distortion leading to truncation of the program, and in turn, low computational accuracy [63, 64]. The issue of mesh distortion is solved by using the Arbitrary Lagrangian–Eulerian (ALE) approach which merges both Lagrangian and Eulerian analysis to redefine the mesh continuously arbitrary as the simulation continues [65, 66]. This allows the mesh to move freely from the material. However, an increase in computational time [54, 65, 67], inaccurate prediction of particle–substrate interface temperature [68], unreasonable particle deformation at high impact velocities [69], and reduction in equivalent plastic strain [70] are some of the limitations of ALE method.

#### The Eulerian method description

Li et al. [33, 66] first employed the Eulerian method for modelling particle impact in CS. In this method, the issue of excessive element distortion encountered in the Lagrangian method is circumvented by regarding the overall mesh as two overlapping meshes: a background mesh that is fixed in space, and a material that can flow through the fixed mesh. Abaqus CAE software offers one the flexibility to assign separate materials to the different regions of the model [71]. Also, the material inside the mesh can flow freely, which makes it sufficient to model the severe plastic deformation experienced in CS [72]. In fact, it is reported that results from the Eulerian method are comparable to those obtained from experimental studies [18, 64, 72–76]. The disadvantages of this method are that there are high simulation run times resulting from the fine mesh and severe plastic deformation. Also, the contact properties such as the coefficient of friction cannot be modified.

#### Coupled Eulerian–Lagrangian (CEL) method description

This method combines the Lagrangian and Eulerian formulations and it is more robust for better simulation results. The CEL simultaneously solves the problems of mesh distortion and unrealistic particle deformation in the Lagrangian method as well as allows for tracking of the interface between the particle and the substrate which is impossible in the pure Eulerian method [77, 78]. For CEL, one part of the model (either particle or substrate) is modelled using the Eulerian formulation, while the other part is described using the Lagrangian formulation [79]. For easy implementation of the Eulerian part of the model, the volume of fluid (VOF) method is used as the foundation for the CEL model. A snag to this method is the inability to trace the history of the change in material behaviour [68].

#### Smoothed-particle hydrodynamics (SPH) description

Smoothed Particle Hydrodynamics is a mesh-free Lagrangian method that can handle/undergo severe plastic deformation [80], making it suitable for modelling CS-impacting processes [33]. In SPH, continuous material is modelled to depict a sequence of particles with some physical characteristics that well describe the properties of the material [81, 82]. Prediction of the ${v}_{\text{cr}}$ in CS can be performed using the SPH technique [82, 83]. Due to the meshless attribute of this method, it can be employed to simulate the multiparticle impact process in CS [83]. However, this method is associated with tensile instability, in addition to low computational accuracy, high computational effort, and time to achieve accurate results [83].

#### Molecular dynamics (MD) method description

MD is a computer simulation method that allows for the prediction of time change of atomic or molecular interactions using Newton’s equations of motion [84, 85]. The basic requirement of MD simulation is that it includes a set of conditions that define the initial locations and velocities of all the particles (atoms), as well as the interaction potential defining the forces in these particles [86]. MD can be used to model the CS process to investigate the microscopic bonding mechanism of the coating and substrate system [87]. The method can be used for simulating structural transformation, grain boundary, defect formation, radiation, elastic/plastic mechanical properties, etc. The Larger-scale Atomic/Molecular Massively Parallel Simulator (LAMMPS) code developed by Sandia National Laboratories was used to simulate the impact of Cu particles on a Cu substrate [88]. An extension of the MD method is the Quasi-coarse-grained dynamics (QCGD); it links the mesoscale gap between classical molecular dynamics and continuum simulations by unfolding the atomic-scale science of particle deformation, jetting, bonding, and microstructure evolution in CS process [89–91]. This method models the thermodynamic and shock response at the mesoscales by coarse-graining the microstructure at the atomic level using representative atoms (*R-atoms*).

#### Important material models in FEA-based approach

Since the principle of CS is built on plastic deformation of the particle and substrate, the selected plasticity model in a finite element analysis affects the predicted deformation pattern in materials in contact. In what follows, we highlight a few material models commonly used for analysing a unit process of CS. Readers interested in other existing models are referred to Ref. [92, 93].

(i) *Johnson–Cook (JC) model* [94]: JC is described in Eq. 1 and it is the most widely used model in numerical simulation of CS. It depicts the plastic response of materials and contemplates strain hardening (first term), strain rate hardening (second term), and thermal softening effects (third term). Although this model is simple to use and mimics most practical situations, the model loses its ability to explain the increase in flow stress at high strain rates beyond a critical value, e.g.10^5^ s^−1^ for Cu [92].
  1 $${\sigma }_{\mathrm{JC}}=\left[A+B{\varepsilon }^{n}\right]\left[1+C{\dot{\mathit{ln}\varepsilon }}^{*}\right]\left[1-{\left(\frac{T-{T}_{\mathrm{room}}}{{T}_{\mathrm{m}}-{T}_{\mathrm{room}}}\right)}^{m}\right]$$
  where $\sigma_{\text{JC}}$ is the flow stress of the material, *A, B, n, C,* and *m* are the material-dependent constants, $\varepsilon$ is the equivalent plastic strain, ${\dot{\varepsilon }}^{*}$ is the equivalent plastic strain rate ($\dot{\varepsilon }$) normalized by a reference strain rate ($\dot{{\varepsilon }_{\text{o}}}$). *T*_room_ and *T*_m_ are the room temperature and melting temperature, respectively.
(ii) *Modified Johnson–Cook model* [95]: This model addresses the problem encountered in the original *JC model*. Here, the flow stress at high strain rate values is accounted for as shown in Eq. 2.
  2 $$ \sigma _{{{\text{JC}}}}  = \left[ {A + B\varepsilon ^{n} } \right]\left[ {1 + Cln\frac{{\dot{\varepsilon }_{{\text{p}}} }}{{\dot{\varepsilon }_{0} }}\left( {\frac{{\dot{\varepsilon }_{{\text{p}}} }}{{\dot{\varepsilon }_{{\text{c}}} }}} \right)^{D} } \right]\left[ {1 - \left( {\frac{{T - T_{{{\text{room}}}} }}{{T_{{\text{m}}}  - T_{{{\text{room}}}} }}} \right)^{m} } \right] $$
  3 $$D=\left\{{}_{x, { \dot{\varepsilon }}_{\text{p}}\ge {\dot{\varepsilon }}_{\text{c}}}{}^{0, {\dot{\varepsilon }}_{\text{p}} < {\dot{\varepsilon }}_{\text{c}}} \,{\text{and}}\, {\dot{\varepsilon }}_{\text{c}}=y{s}^{-1}\right.$$
  where *D* is a parameter that is non-zero ($x$) when the plastic strain rate, ${\dot{\varepsilon }}_{\text{p}}$, is equal to or greater than the critical strain rate, $\dot{\varepsilon }_{\text{c}}$ ($y$). $\dot{\varepsilon }_{0}$ is the reference strain rate.
(iii) *Preston-Tonks-Wallace model* [96]*:* This model is based on the mechanical threshold stress derivation and it is for metallic plastic flow under high velocity impacts. The flow stress in this model is given by
  4 $$\sigma =2\left[{\widetilde{\tau }}_{\text{s}}+\alpha ln\left[1-\varphi {\text{exp}} \left(-\delta -\frac{\theta \varepsilon }{\alpha \varphi }\right)\right]\right]{G}_{\text{P}}$$
  5 $$\alpha =\frac{{S}_{0}-{\widetilde{\tau }}_{\text{y}}}{{P}_{\text{PTW}}}, \delta =\frac{{\widetilde{\tau }}_{\text{s}}-{\widetilde{\tau }}_{\text{y}}}{\alpha }, \varphi =\mathrm{exp}\left(\delta \right)-1$$
  where ${\widetilde{\tau }}_{\text{s}}$ is the normalized work-hardening saturation stress,$s_{0}$ is the saturation stress at 0 K, ${\widetilde{\tau }}_{\text{y}}$ is the normalized yield stress, $\theta$ is strain hardening rate, *ε* is the equivalent plastic strain, ${P}_{\text{PTW}}$ is the strain hardening constant, and ${G}_{\text{P}}$ is the plastic shear modulus.
(iv) Zerilli-Armstrong (ZA) model: This model is capable of accounting for the plastic behaviour of materials as it depicts the flow stress at high temperatures [97, 98]. The flow stress is given by the equation;
  6 $$\sigma =\left({C}_{1}+{C}_{2}{\varepsilon }^{n}\right)\mathrm{exp}\left\{-\left(\left({C}_{3}+{C}_{4}{T}^{*}\right){T}^{*}+\left({C}_{5}+{C}_{6}{T}^{*}\right)ln{\dot{\varepsilon }}^{*}\right)\right\}$$
  7 $${T}^{*}=T-{T}_{r}$$

where *C*_1_, *C*_2_, *C*_3_, *C*_4_*, C*_5_, *C*_6_, and *n* are material constants, *ε* is the equivalent plastic strain, $\dot{\varepsilon }^{*}$ is the equivalent plastic strain rate normalized with respect to the reference strain rate, $\left( {\frac{{\dot{\varepsilon }_{{\text{p}}} }}{{\dot{\varepsilon }_{0} }}} \right)$, *T* and *T*_r_ are the absolute and reference temperatures, respectively.

### Experimental methods

#### Wipe test for “single” multi-microparticle impact

**Figure 3:**
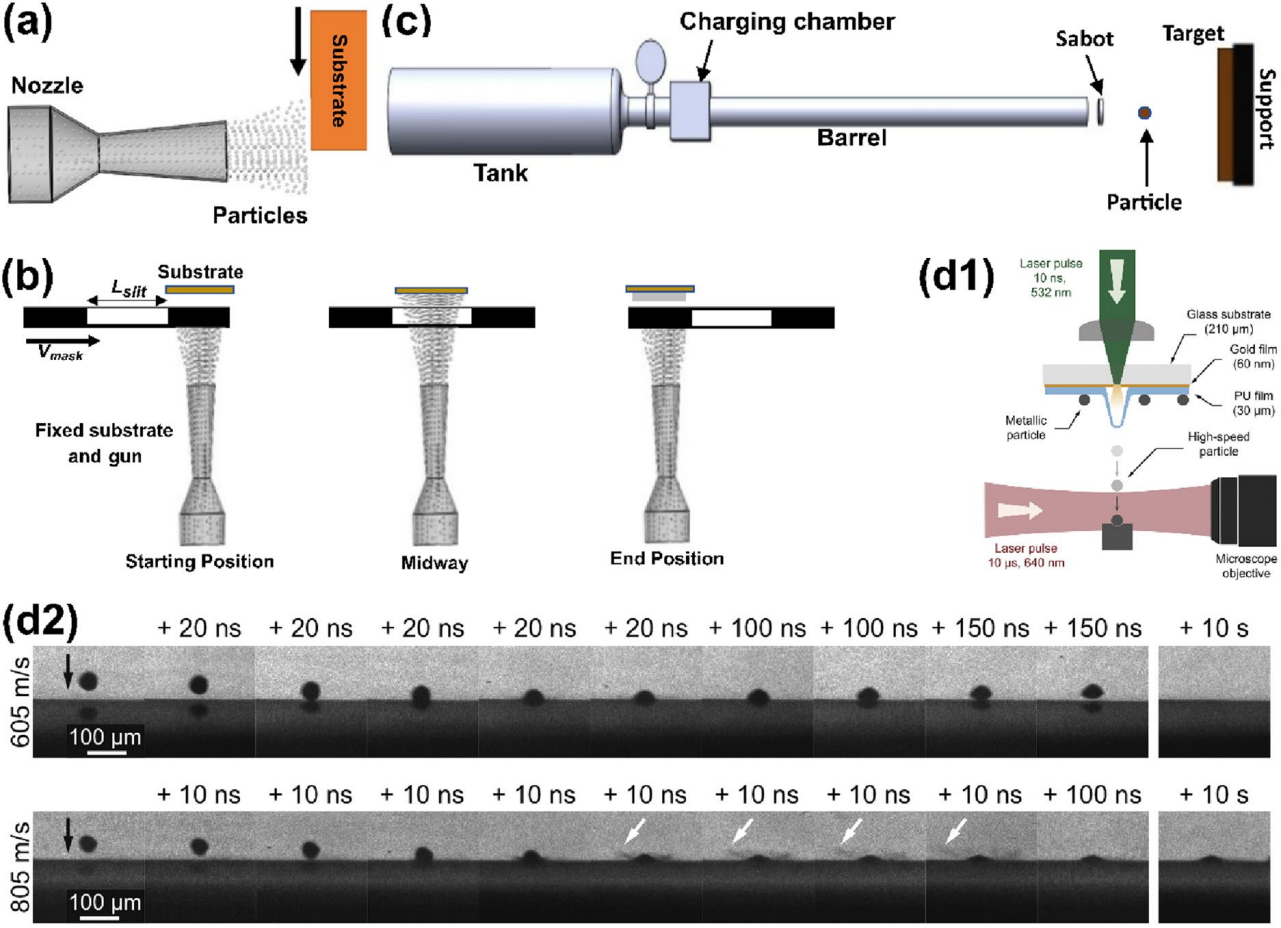
Schematic representation of the experimental single-particle impact set-ups: (a) the wipe test for “single” microparticle impact; (b) masking slit-type variant of the wipe test (redrawn from Ref. [101]); (c) the ballistic airgun for macroparticle impact (redrawn from Ref. [103]), and (d) the Laser-induced particle impact tester, LIPIT, and its corresponding typical multiframe sequences showing real-time observation of 45-μm Al particle impacting an Al substrate at 605 m/s (rebound) and 805 m/s (bonding), reprinted with permission from Ref. [104], copyright 2015 Elsevier.

The wipe test is a technique for studying the morphology of deposits/splats of a single *multi-microparticle* impact process [23, 99, 100]. To obtain individual particles on a substrate, the prepared substrate is swiftly passed through a fixed spray jet [45, 59] as shown in Fig. 3(a). To avoid velocity overlapping and the problem of identifying a particle position in a diverging beam, a variant of the wipe test was proposed by Guetta et al. [101]; the variant uses a mask between the stationary gun and substrate [Fig. 3(b)]. By moving the mask so that a slit comes in line with the particle jet, particle deposition occurs on the substrate. The limitations of the wipe test technique are its inability to measure the exact temperature and velocity of the individual particles during impact [29]. The technique also misses possible particle interaction details, and in turn, makes it difficult to distinguish particles that arrive at the substrate along a straight trajectory from those that are deposited ahead or behind [102].

#### Ballistic airgun for “macroparticle” impact

In this approach, a ballistic airgun is used to impact a single macroparticle on the substrate as shown in Fig. 3(c). Here, the geometry, dimensions, and weight of the particle are defined to depict the nature of the powder particles being investigated [103]. A high-frequency camera is used for tracking, observing, and measuring the particle impact and rebound speeds. One disadvantage of this approach is that the physical and microstructural evolutions in ball-particles may not reflect actual evolutions during CS processing with more confined particle volume: the powder particles in CS (10–100 µm) are far smaller and lighter than the macroparticle balls (1.5–20 mm in diameter) used in this technique [29, 103].

#### Laser-induced particle impact test (LIPIT) for single microparticle impact

To address the setbacks in the conventional single-particle characterization methods such as the wipe test and ballistic airgun, recent studies [25, 104, 105] employed an advanced technique (LIPIT) developed by Lee et al. [106] and upgraded by Veysset et al. [105]. The LIPIT allows the real-time observations of single microparticle impact moment at a micron length scale and nanoseconds time scale. An excitation laser pulse is directed towards a launching pad assembly (usually made up of a glass substrate, gold film, polyurea film) on which the particles are spread. The ablation of the gold film and the subsequent expansion of the polyurea film launches the pre-selected single metallic particle towards the oppositely positioned target/substrate. As part of the LIPIT set-up, a high-frame-rate camera and a quasi-clockwise laser imaging pulse for illumination are positioned near the target to provide real-time data on the impact process. A complete schematic of the LIPIT set-up and the typical multiframe that shows real-time observation of microparticle impact moments and jetting (indicated by white arrows) is shown in Fig. 3(d). More details on the operating principles of LIPIT can be found in Refs. [104–106].

## New insights on bonding in cold spray process

### Impact cases, impact modes, and bonding types

**Figure 4:**
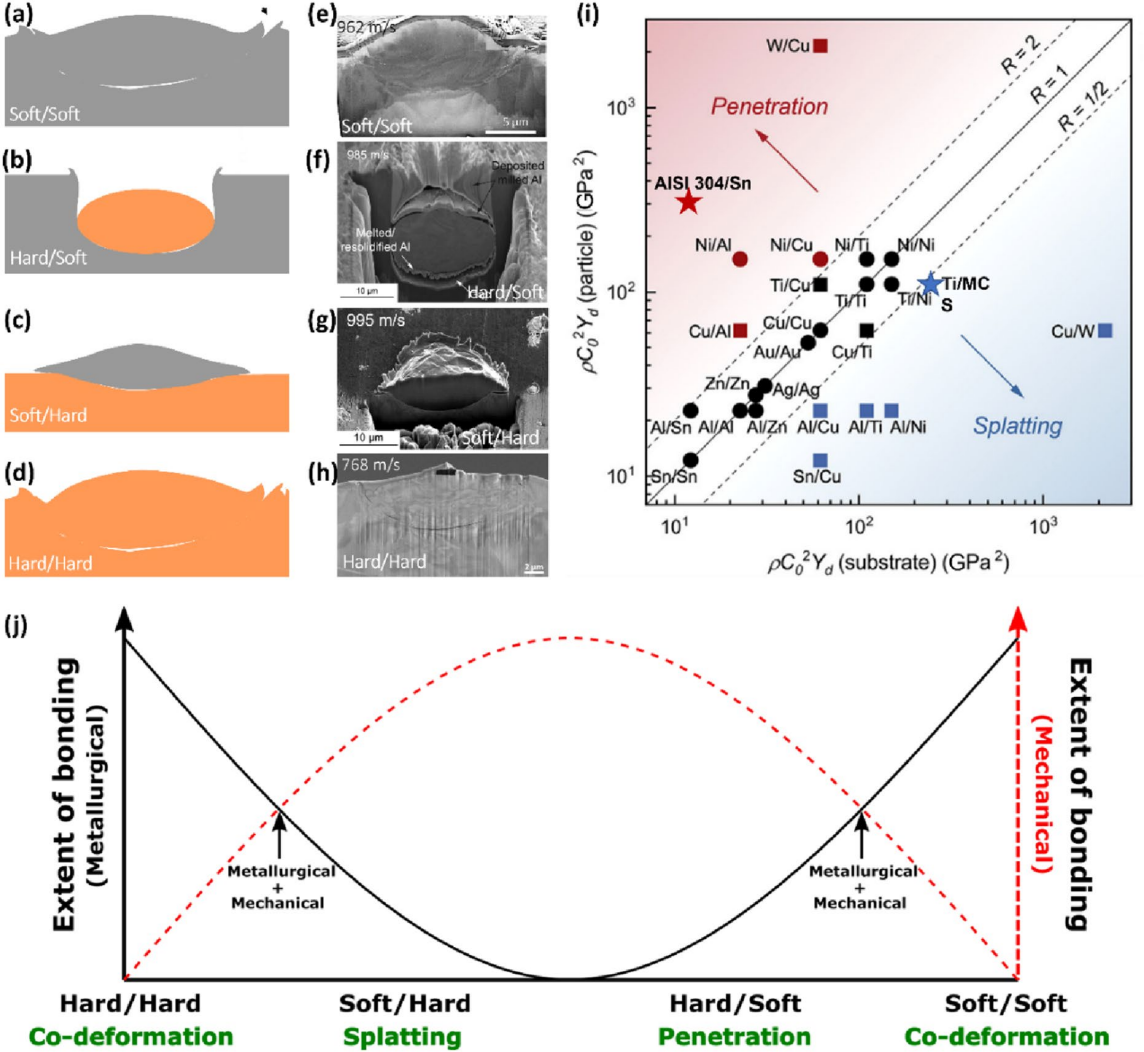
(a–d) Schematic and (e–h) experimental cross-section SEM images showing impact modes in different particle/substrate configurations for Al and Cu; e, h, reprinted with permission from Ref. [26], copyright 2020 Elsevier, f, g, reprinted with permission from Ref. [107], copyright 2020 Elsevier; (a, e and d, h) co-deformation, (b, f) penetration, and (c, g) splatting; (i) impact modes predictive map for similar and dissimilar pure metals (square and circular data points), and now showing its accurate prediction of impact mode for alloys (starred data points from Refs. [108, 109]), reprinted with permission from Ref. [107], copyright 2020 Elsevier; and (j) conceived connection between bonding types, impact cases (particle/substrate configurations), and impact modes.

In CS processing, particles’ response to high velocity impact is a function of the material properties such as density and dynamic yield strength [6]. Such impact results in extreme plastic deformation of the particles which thus informs the extent of flattening, and in turn bonding [25, 26]. Generally, there exist four possible configurations in any *impact case*—soft/soft (similar ductility or hardness), hard/soft (particle is harder than the substrate), soft/hard (particle is more ductile than the substrate), and hard/hard (similar ductility or hardness), as schematized in Fig. 4(a–d). These *impact cases* have been numerically examined by Bae et al. [54], in which co-deformation was observed in soft/soft and hard/hard configurations, although less extensive deformation in the latter. An example of different material combinations for Al (soft) and Cu (hard) is shown in Fig. 4(e–h). These combinations result in different *impact modes*: co-deformation (Al/Al and Cu/Cu), penetration (Cu/Al), and splatting (Al/Cu). We note here that the use of “hard” or “soft” for different impact scenarios should be relative depending on the constituting material type. For instance, Cu is “hard” in Al and Cu combinations [Fig. 4(e–h)], but Cu is “soft” in Cu and W combinations [107].

Although the “hard” or “soft” classification is not that trivial as there have been instances of misinterpretation of impact cases and impact modes. For instance, a Ni/Cu impact is considered a soft/soft impact case [60, 54] despite suggesting a hard/soft configuration. This begs for a clearer understanding of impact cases and their connection to impact modes, without ambiguity. Thanks to the capabilities of LIPIT, Hassani et al. [107] recently examined multiple site-specific single microparticle impact sites and cross-sections, and further developed a theoretical framework for predicting impact modes in any impact cases [Fig. 4(i)]. The authors proposed materials property-based impact ratio, $R=\frac{{(\rho {C}_{\text{o}}^{2}{Y}_{\text{d}})}_{\text{p}}}{{(\rho {C}_{\text{o}}^{2}{Y}_{\text{d}})}_{\text{s}}}$, where $\rho {, C}_{\text{o}},{Y}_{\text{d}}$ are density, shock velocity, and dynamic yield strength, that successfully predict dominant impact modes—co-deformation, splatting, and penetration, when $R$ is close to, much less than, and much greater than 1, respectively [107]. Although the predictive map in Fig. 4(i) was used for similar and dissimilar pure metals, we show here that the map can accurately predict impact modes for impact cases involving alloys [see starred datapoints of AISI 304/Sn [108] and Ti/MCS [109] in Fig. 4(i)].

Irrespective of the impact cases and modes, two types of bonding sets on—metallurgical (chemical) and mechanical (physical) bonding [6, 25]; metallurgical bonding involves atomic interaction of two clean metallic surfaces in contact to initiate metallic bonding, while particles embed or anchor into the substrate without a need for chemical interaction in the mechanical bonding case [110]. Because the word “mechanism” implies an established process by which a phenomenon takes place, we strictly reserve “metallurgical” and “mechanical” as distinct *bonding types*, and not as *bonding mechanisms*, as they have been confusingly used. The two bonding types can co-exist or occur singly in a single microparticle impact depending on the materials’ properties and particle/substrate configuration.

*Subsection summary* Assessing several single microparticle impact experiments [25, 26, 107, 108, 111], the connection between impact cases, impact modes, and bonding types can be envisioned for the first time in Fig. 4(j). It should be noted that the hard/hard impact case in Fig. 4(j) does not contemplate elastic -particle and -substrate materials in which most of the impact kinetic energy is recovered upon impact, i.e. the coefficient of restitution (ratio of rebound velocity to impact velocity) tends to 1, but rather materials that undergo “reasonable” plastic deformation when in contact. Although metallurgical and mechanical bondings are on the extremes of Fig. 4(j), there exists a cross-over point where both bonding types co-exist which has been experimentally validated, e.g. in Cu/Al impact case [101].

### Barriers to bonding: extrinsic and intrinsic

**Figure 5:**
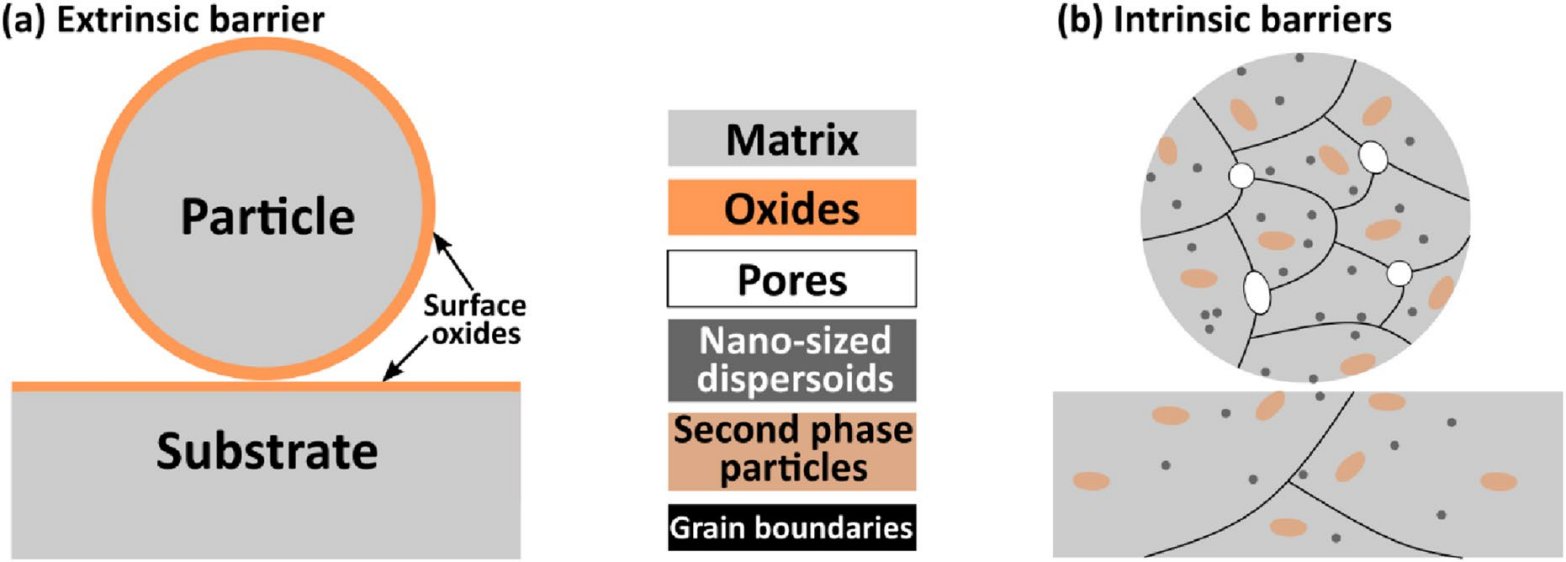
Schematic diagrams showing common (a) extrinsic and (b) intrinsic barriers to bonding during cold spray process.

**Figure 6:**
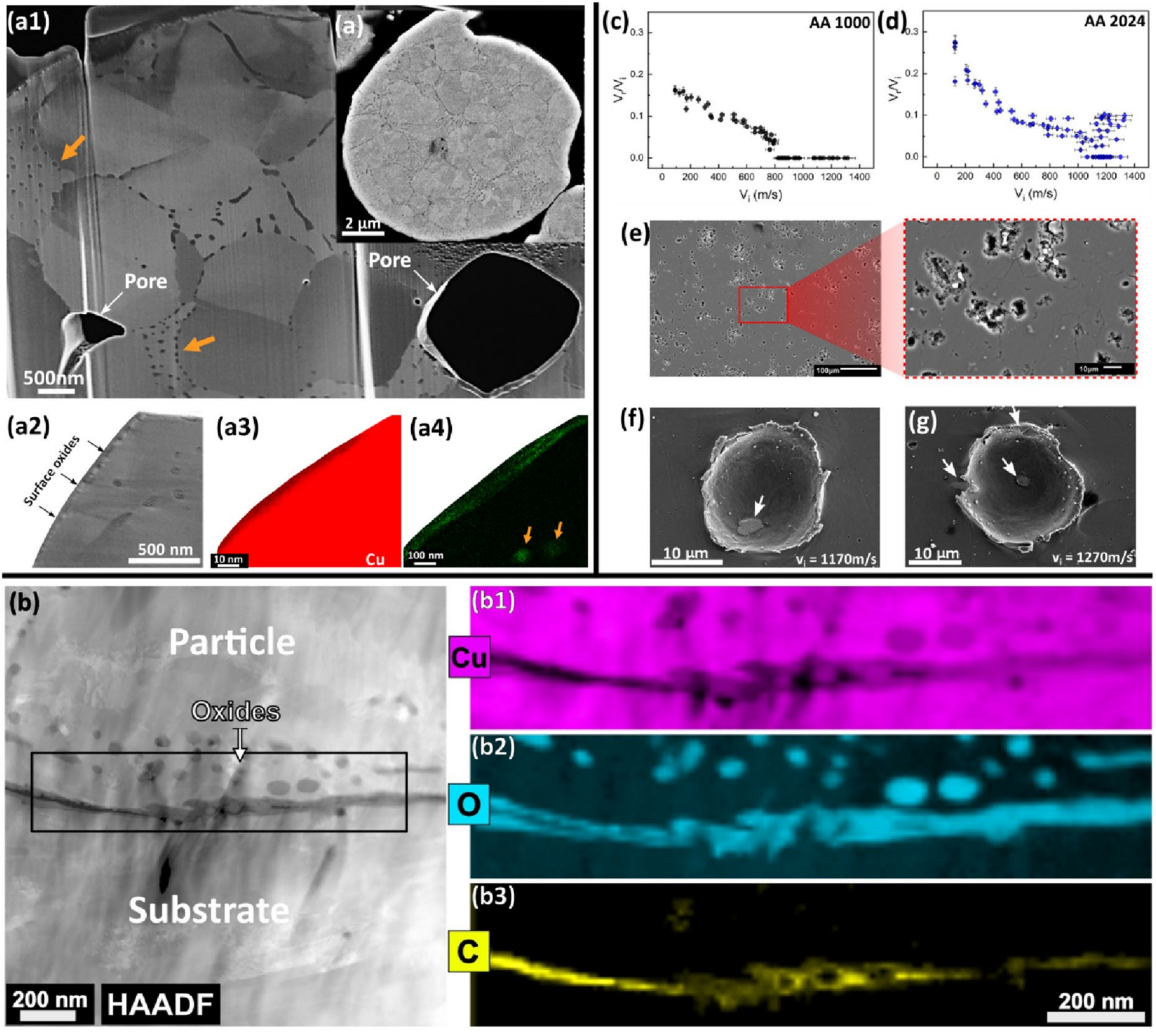
(a) Cross-sectional SEM/STEM micrographs and EDS maps of undeformed Cu particles showing surface oxide, pores, and nano-sized dispersoids (orange arrows), Adapted with permission from Ref. [50], copyright 2021 Elsevier; (b) the ADF STEM images and EELS maps of a Cu particle/substrate interface showing the presence of surface oxides, spherical nano-dispersoids, and Carbon, reprinted with permission from Ref. [114], copyright 2022 Elsevier; (c, d) the coefficient of restitution, ratio of the rebound and impact velocity, as a function of the ${v}_{\text{i}}$ for 14 micron-sized aluminium (AA 1000) and aluminium alloy (AA 2024) particles, reprinted with permission from Ref. [52], copyright 2019 Elsevier; and (e) SEM micrographs of etched undeformed AA 2024 alloy that shows second-phase particle distribution. (f, g) SEM images showing micron-sized second-phase particles (white arrows) within and at the rim of the crater formed when AA 2024 spherical particle impacts AA 2024 substrate.

Despite the many debates on the mechanism of bonding, there is a consensus that bonding will set on when clean metal surfaces are in contact at sufficient ${v}_{\text{i}}$. Therefore, any features that hinder the development of clean metal–metal contact or any phenomenon that consumes part or all the energy budget to initiate metal–metal contact are regarded as *barriers to bonding*. We classify these barriers as *extrinsic* and *intrinsic* depending on how they interact with the surfaces in contact. The *extrinsic* barrier, which is the most studied, is external barriers such as native surface oxides on both the particle and substrate as shown in Figs. 5(a) and 6(a). Previous studies have shown that the presence of surface oxides on both the particles [112] and substrate [113] increases ${v}_{\text{cr}}$. In a recent single microparticle impact study by Lienhard et al. [112], the effect of oxide thickness on ${v}_{\text{cr}}$ was experimentally quantified: with just ~ 60% increase in oxide thickness from 5 nm in the as-received state, ${v}_{\text{cr}}$ of aluminium microparticles increased by more than 100 m/s. Recent works also show the presence of amorphous carbon on Cu particle surface [50, 114]; it is believed that carbon possibly originated from contamination during particle production, handling, and storage, and it provides additional barriers to metallurgical bonding [114].

Meanwhile, the *intrinsic* barriers are constituting microstructural features that can be present in both the particle and substrate materials; they include, but are not limited to, micropores, oxide dispersoids, and micron-sized second-phase particles (SPP), as shown in Figs. 5(b) and 6(a). The roles of *intrinsic* barriers are not well studied, but some of their contributions to the onset of particle bonding were recently revealed in single microparticle impact studies. Using hydrocode modelling of two impacting spheres, Davison et al. [115] observed that pores in materials expend a large amount of energy to close “pore space”. This leads to a dramatic increase in the amount of heating and the attenuation of shock energy during impact. Although the contribution of pores to bonding may be minimal, this phenomenon should consume some fraction of the energy budget for bonding. In a separate study [114] [Fig. 6(b)], nano-sized spherical oxide dispersoids within the Cu matrix, but close to the particle surface, were expelled during impact to form an additional barrier to bonding at the particle–substrate.

An important intrinsic barrier to bonding that is yet to be rigorously explored is the presence of micron-sized SPPs, mostly in alloys. The work of Hassani et al. [52] possibly hints at their role as shown in Fig. 6(c, d): while the onset of bonding can be clearly discerned when CoR turns to zero for Al/Al impact case, scattered data points (mixture of bonding and rebounding) exist for AA 2024/AA 2024 at high velocities. To put this in proper perspective, an etched AA 2024 alloy substrate showing the distribution of SPPs is presented in Fig. 6(e); these micrographs show that SPPs can be ubiquitous so that the onset of bonding becomes a probability depending on the local microstructural characteristics of the launched microparticle surface and the substrate. Figure 6(f, g) shows samples of impact sites where particles launched at 1170 and 1270 m/s rebounded; the figures show clear evidence of micron-sized SPPs in and at the rim of the craters. SPPs are strengthening sources in alloys, but they are hard, and brittle compared to the matrix. This can result in localized variation in spall strength, and in turn, the mixture of bonding and rebounding cases as seen in Fig. 6(d).

*Subsection summary on barriers to bonding* It is clear that to initiate bonding, particle kinetic energy must be sufficient to overcome these barriers. However, not all these barriers are present in all materials systems, making it difficult to suggest a single mechanism that explains all the impact bonding processes. In what follows, we present diverse bonding mechanisms by examining the phenomena that set on prior to or during bonding, most of which are occasioned by the presence of any of the aforementioned barriers.

### Evolved phenomena during single microparticle impact and bonding mechanisms

As a next step, we examine the recent works on the phenomena that set on during particle impact to develop clean metal surfaces that aid metallurgical bonding.

#### Oxide layer breakup and delamination

**Figure 7:**
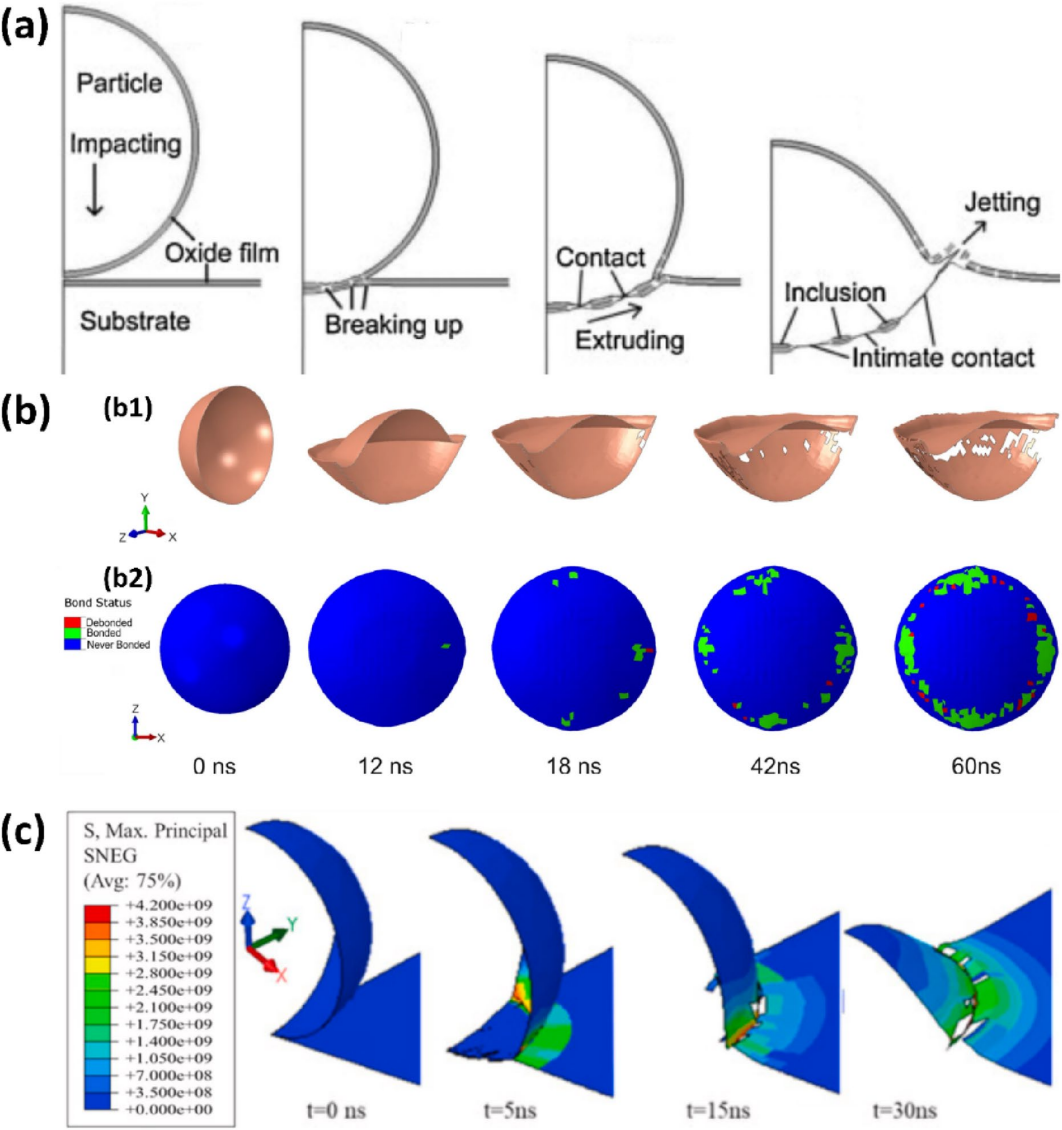
(a) Schematic of oxide breakup mechanism, reprinted with permission from Ref. [65], copyright 2009 Elsevier; (b) FEM modelling of Cu surface oxide deformation and breakup, and bonding element status on the particle side during the deposition process, reprinted with permission from Ref. [119], copyright 2021 Springer Nature; and (c) Maximum principal stress in FEM simulation of Al surface oxide, reprinted with permission from Ref. [120], copyright 2022 Elsevier.

As microparticle impresses into a substrate during high velocity impact, the breakup of surface oxides, if present, is the first physical phenomenon that sets on. This is because stresses on oxide scales are applied through the particle or substrate core metal; hence, oxides crack once the metallic core strains beyond yield, i.e. $\varepsilon >{\varepsilon }_{\text{m}}=\frac{{\sigma }_{\text{m}}}{{E}_{\text{m}}}$ (${\sigma }_{\text{m}}$ and ${E}_{\text{m}}$ are yield stress and Young’s modulus of the metallic core, respectively), to form a series of discontinuous oxide islands, but still attached to the particle or substrate metal [116]. This process is termed oxide layer breakup in the field of oxide fracture mechanics [116], yet the same terminology is a long-standing existing mechanism used to describe the breaking and expulsion (not *extrusion* as commonly used) of oxides from the particle–substrate interface to initiate bonding in cold spray process as schematized in Fig. 7(a).

The indirect evidence of oxide layer breakup mechanism in Fig. 7(a) are in most cases inferred from wipe test experiments [117, 118] and numerical modelling [65]. A recent numerical study on the role of surface oxide layer on Cu particle–substrate adhesion shows local oxide layer removal that produces an oxide-free ring-like region somewhere between the particle rim and the south pole [119]. Meanwhile, the oxide shell remained nearly intact in the south pole where the shear stress is minimum, as shown in Fig. 7(b). Navabi et al. [120] combined MD and FEM simulations to model the fate of surface oxides in a 6061 Al-6061 Al impact combination; the MD simulation was used to obtain mechanical properties of oxide films that were subsequently incorporated into FEM modelling of single microparticle impacts. The authors found that shear stresses near the particle–substrate periphery promote the cracking of oxide layers and expulsion of the splats [120].

However, an increase in oxide thickness implies a lower applied stress requirement for oxide cracking due to the propensity of finding longer cracks or discrete cavities with interacting stress fields in thicker oxides [121]. In other words, thicker oxides would require lesser strain or energy to break than a thinner oxide [116], and as such, an increase in oxide thickness should intuitively result in lower ${v}_{\text{cr}}$ for particle adhesion in the CS process. However, the exact opposite is observed: thicker oxide layer results in higher ${v}_{\text{cr}}$. This implies that a complementary energy-consuming process beyond oxide breakup/cracking must be involved in the development of clean metal–metal contact.

**Figure 8:**
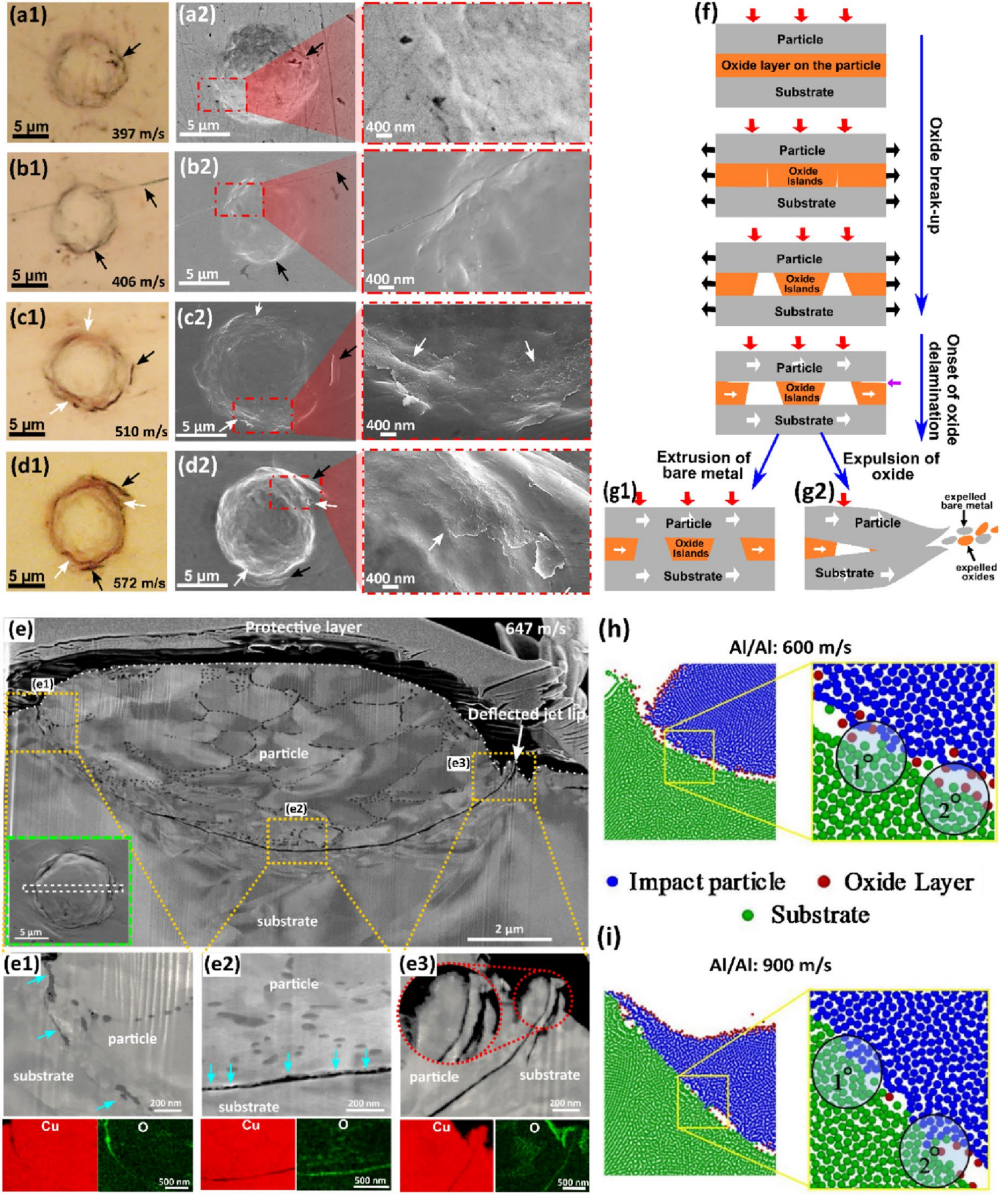
(a1–d1) Optical and (a2–d2) corresponding SEM micrographs of impact sites where particle hits the substrate at (a) 397, (b) 406, (c) 510, and (d) 572 ms^−1^, using LIPIT [50], reprinted with permission from Ref. [50], copyright 2022 Elsevier: the delaminated oxides are indicated by white arrows, while black arrows point at possible artifacts from polishing steps and elevated regions due to the jetting process; (e) Cross-sectional SEM micrographs and STEM-EDS maps of permanently adhered particle launched at 647 ms^−1^: it shows evidence of bare metal extrusion into the gaps between trapped delaminated oxides or oxide islands in (e1), reprinted with permission from Ref. [50], copyright 2022 Elsevier. (f, g) Schematic diagram summarizing the process of oxide breakup and delamination, and the resulting bonding [50], reprinted with permission from Ref. [50], copyright 2022 Elsevier; (h, i) SPH mesh-free-based simulated interfaces of impacted particles at 600 and (d) 900 ms^−1^, respectively [122], reprinted with permission from Ref. [122], copyright 2021 Elsevier.

**Figure 9:**
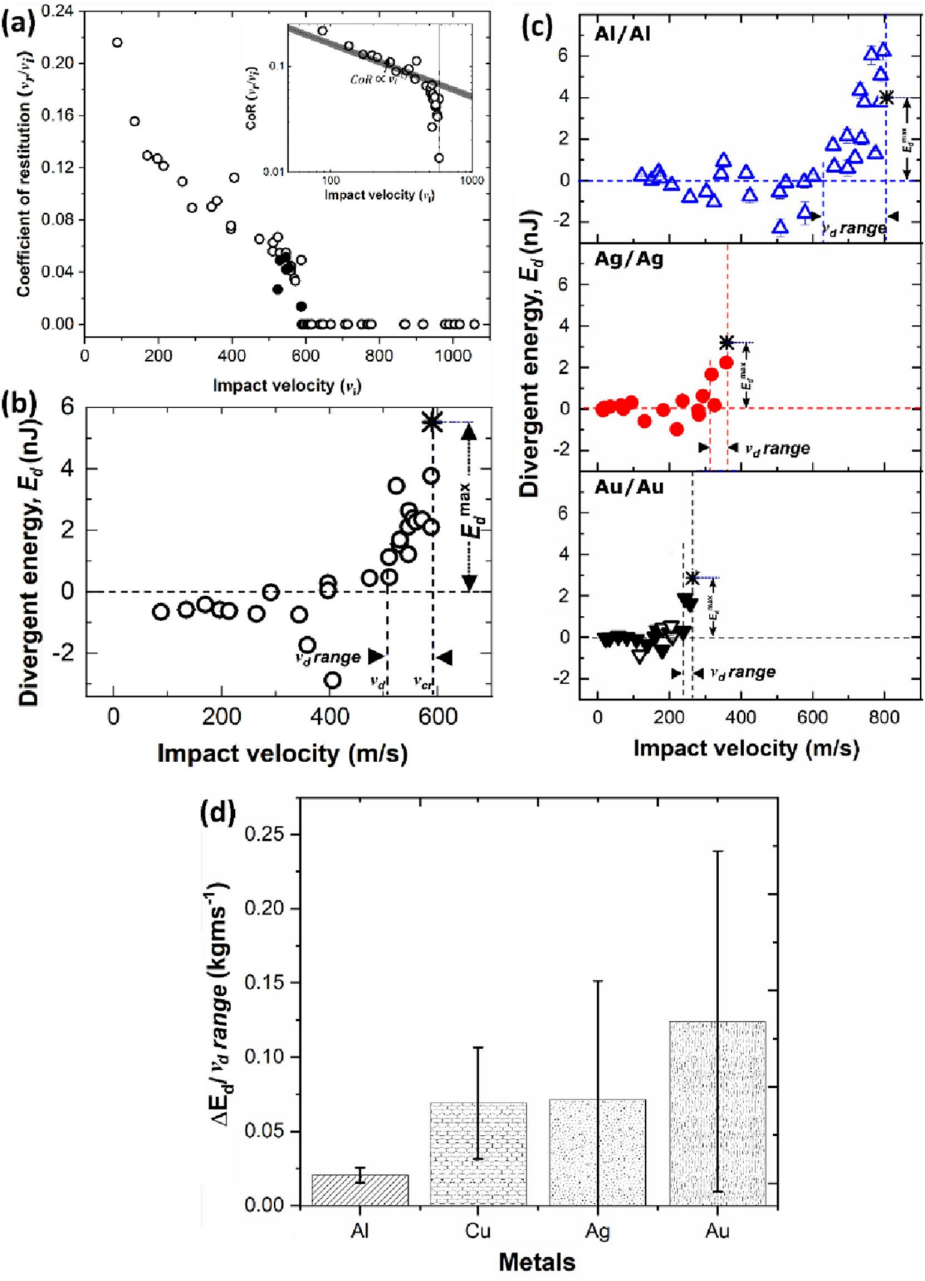
(a) Coefficient of restitution *versus* ${v}_{\text{i}}$ plot; (b) Divergent energy (determined as the difference between the rebound kinetic energy and the power-law predicted energy) against impact velocity plot, (a) and (b) reprinted with permission from Ref. [50], copyright 2022 Elsevier; (c) Divergent energy (determined as the difference between the rebound kinetic energy and the power-law predicted energy) *versus* ${v}_{\text{i}}$ plot for similar impact cases (Al/Al, Ag/Ag, and Au/Au), reprinted with permission from Ref. [124], copyright 2019 Elsevier; and (d) bonding “momentum”, the ratio of divergent energy and divergent velocity against different metals, showing how the rate of bonding increases with increased metal nobility.

In a site-specific study of single Cu microparticle impacting a mirror-polished Cu substrate, Tiamiyu et al. [50] report the first evidence of oxide layer delamination in the regime where particle rebounds: surface oxide layer on particles start to delaminate at impact velocities (~ 510 m/s) where jetting of the substrate also sets on around the periphery of the crater [Fig. 8(a–d)]. The authors showed that the delamination process consumed ~ 30% of the divergent energy (${E}_{\text{d}}$)—energy expended beyond plasticity, which is the difference between the energy predicted by the power-law and the measured rebound kinetic energy [123], as shown in Fig. 9(a, b). This observation supports the criteria of oxide failure in terms of how they propagate or delaminate rather than how through-cracks are formed [116]*.* We, therefore, believe the well-known oxide breakup misses the very crucial aspect of the expulsion process—*oxide delamination*, which in essence is the main energy-consuming process.

A notable observation from this work [50] is the evidence of bare metal extrusion into the gaps between trapped delaminated oxides or oxide islands that are either still attached to the particle due to insufficient jetting [Fig. 8(e)]. Cataloguing the observations from the classical oxide breakup mechanism and the new evidence of oxide layer delamination provides a complete oxide-based bonding mechanism that can explain the formation of metallurgical bonding with insufficient [Fig. 8(f and g1)] and sufficient [Fig. 8(f and g2)] jetting at the particle–substrate interface. An increase in ${v}_{\text{i}}$ results in more significant jetting, and in turn, cleaner metal–metal contact, as also validated in the SPH numerical modelling of Al/Al impacts in Fig. 8(h and i).

While oxide layer delamination is experimentally confirmed and also energetically favoured to occur in Cu/Cu impacts, the question is whether or not the maximum divergent energy, ${E}_{\text{d}}^{\text{max}}$ (the divergent energy for an impact at velocity ${v}_{\text{cr}}$ and zero rebound velocity) and divergent velocity range, ${v}_{\text{d}}$ *range* (difference between ${v}_{\text{d}}$ and ${v}_{\text{cr}}$) can be used to determine the nobility of material, and in turn, affect the onset of bonding. If indeed oxide layer delamination occur within ${v}_{\text{d}}$ *range*, an increase in ${E}_{\text{d}}^{\text{max}}$/${v}_{\text{d}}$ *range* as metals become noble should be intuitively correct. This is exactly what is observed in Fig. 9(d): the ${E}_{\text{d}}^{\text{max}}$/${v}_{\text{d}}$ *range* for Al/Al, Cu/Cu, Ag/Ag, and Au/Au are 0.021, 0.069, 0.071, and 0.124 kg/ms, respectively, in the order of increasing metal nobility in Fig. 9(b and c). These results show that in a more noble metal like Au, excess energy, ${E}_{\text{d}}$, is mostly consumed by jetting alone so that a clean contact is achieved at much lower ${v}_{\text{cr}}$, as shown in Fig. 9(c). Similarly, in a less noble metal where substantial surface oxide layer is present, dissipated energy for jetting must be high enough to delaminate and expel the oxides first, resulting in higher ${v}_{\text{cr}}$. The ${E}_{\text{d}}^{\text{max}}$/${v}_{\text{d}}$ *range* in Fig. 9(d) can therefore be a useful parameter—somewhat a *bonding momentum—*that indicates the “rate” at which bonding sets on. Note that Fig. 9(d) does not contemplate oxide thickness, and as a direction for future studies, it will be beneficial for the cold spray community to understand more clearly how the variation in oxide thickness influences ${E}_{\text{d}}^{\text{max}}$*,* ${v}_{\text{d}}$ *range,* and ${v}_{\text{cr}}$*.*

#### Adiabatic shear instability and jetting

##### Jetting—adiabatic shear instability vs a natural hydrodynamic effect

**Figure 10:**
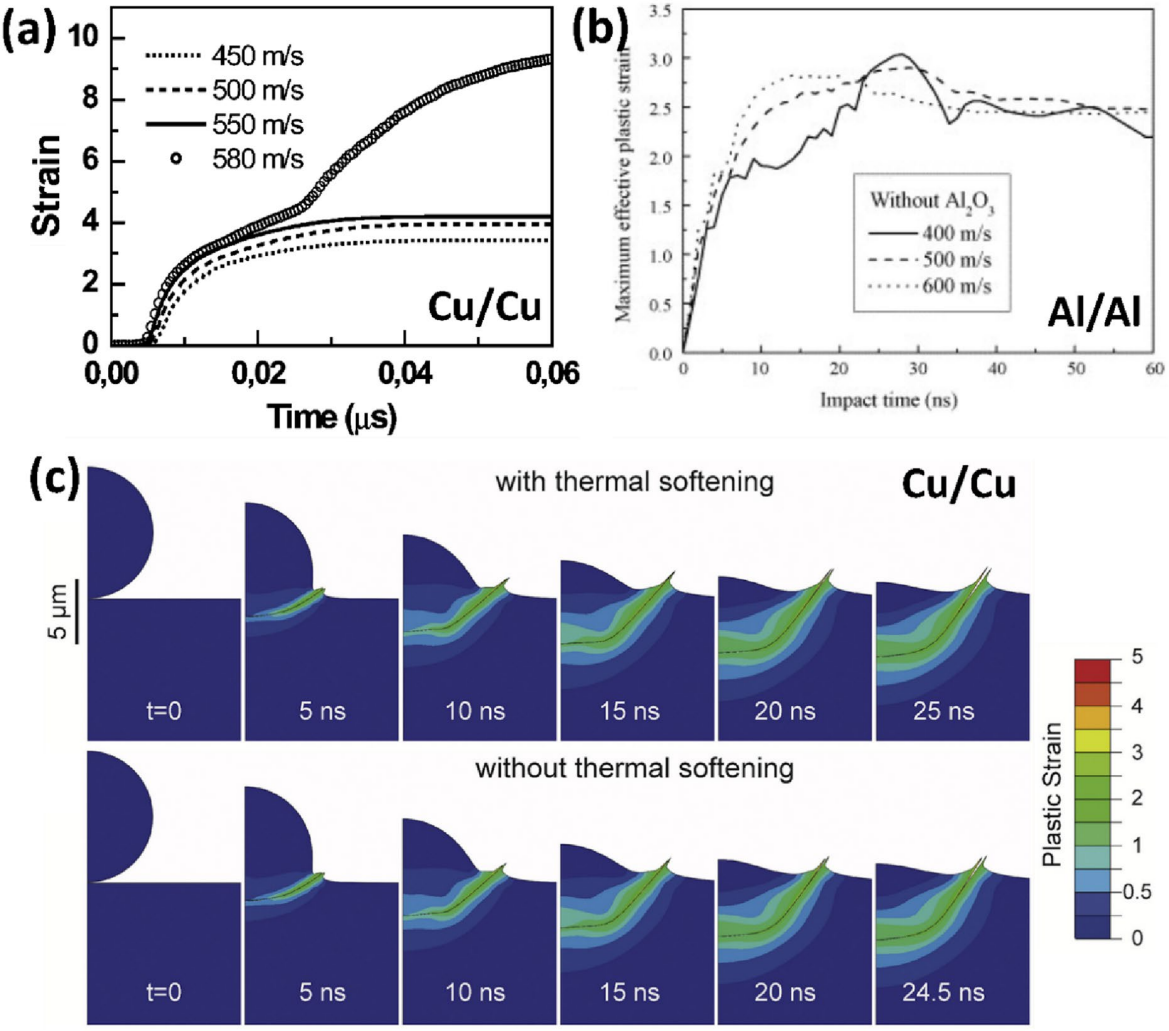
(a) Evolution of plastic strain with time in single Cu/Cu microparticle impact, reprinted with permission from Ref. [67], copyright 2003 Elsevier; (b) Temporal development of maximum effective plastic strain under different particle velocities modelled with and without oxide film, reprinted with permission from Ref. [128], copyright 2007 Elsevier; (c) Von Mises Plastic strain distribution and snapshots of deformation until the end of penetration for material with and without the thermal softening capability, to justify the presence of jetting in both conditions, reprinted with permission from Ref. [51], copyright 2018 Elsevier.

During single microparticle impact, extreme strain and temperature are localized in a narrow region near the particle–substrate interface. The temperature change coupled with excessive plastic strain rate results in local heating. As strain, strain rate, and temperature increase, a point of plastic flow thermal instability known as Adiabatic Shear Instability (ASI) is reached at the interface [125]. ASI is an unstable phenomenon in materials at high strain rates in which strain hardening, strain rate hardening, and thermal softening compete, and the first two effects are overcome by thermal softening [126]. Assadi et al. [67] were the first to point out that the velocity at which ASI sets on during microparticle impact, ${v}_{\text{ASI}}$, as indicated by a jump in strain from ~ 4 to 10 in Fig. 10(a), can be approximated to ${v}_{\text{cr}}$; and by extension, that ASI *contribute/play a role* in bonding [127]. Since then, many (*not explicitly by Assadi *et al*.* [67]) have adjudged ASI as a mechanism for bonding, rather than “contributing” to bonding.

**Figure 11:**
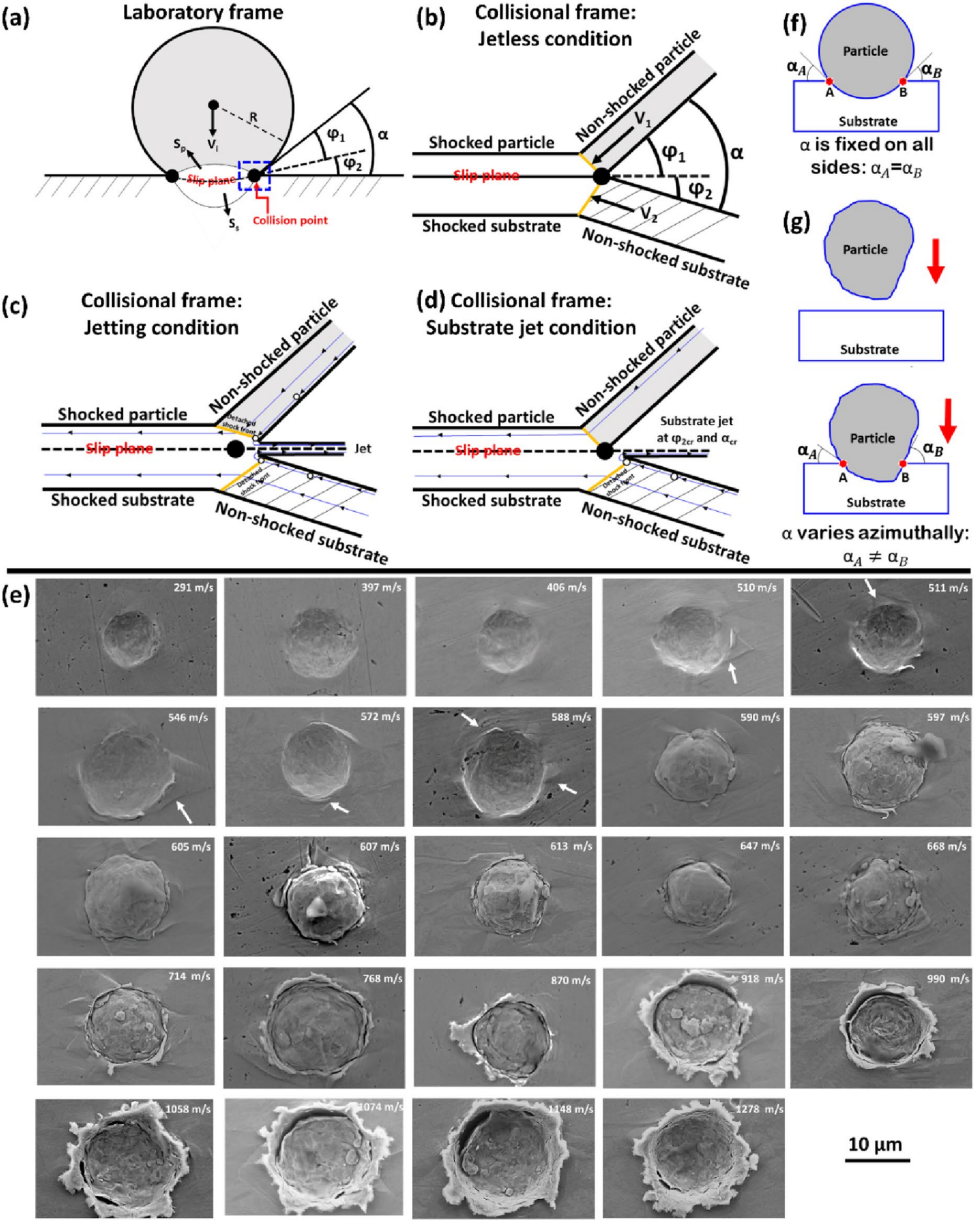
Schematic diagram of the jet evolution theory for (a) the laboratory frame set-up (b) collision frame for jetless condition, (c) collision frame for jetting condition, and (d) collision frame for substrate jetting condition, reprinted from Ref. [129] under the terms of the Creative Commons Attribution-NonCommercial-NoDerivatives 4.0 International License; (e) SEM micrographs of impact sites as Cu particle impact Cu substrate at different impact velocities, reprinted with permission from Ref. [25], copyright 2021 Elsevier. In (a–d, f, g), ${S}_{\text{p}}$ and ${S}_{\text{s}}$ are shock wave in particle and substrate, respectively, $\mathrm{\varphi }$ is the deflection angle, and α is impact angle or wedge angle. The white arrows in (e) point at jetting and shear offsets around the craters.

The debate on whether ASI is necessary for jetting (and adhesion) to occur or not has recently been ignited [51, 52, 65, 67, 127]. Li et al. [65, 128] were the first to point out that the correlation of the “strain jump” in Fig. 10(a) to the onset of ASI in a *Lagrangian-based* numerical simulation was faulty. They argued that no such steep change in plastic strain was observed for Al/Al impact case using the ALE method [128] as shown in Fig. 10(b); hence concluding that the plastic strain jump in Fig. 10(a) was due to the abnormal element distortion that is characteristic of the Lagrangian method. However, this argument may be faulty in itself as the assessed ${v}_{\text{i}}$ in Ref. [128] (400–600 m/s) is much lower than the typical ${v}_{\text{cr}}$ for Al/Al (797–824 m/s [104]); the same equivalent regime (450–550 m/s) where Assadi et al. observed no steep change for Cu/Cu impact case. Also recently, Hassani et al. [51, 52] posit that ASI is not necessary for jetting and in turn, adhesion; their argument was based on the persistent evolution of jetting whether the thermal softening term in the constitutive model in Eq. 1 is “turned on or off” [Fig. 10(c)]. They also observed in situ jetting during impact where bonding occurred. Hassani et al. further backed their argument by considering the jet evolution theory such as schematized in Fig. 11(a–c) [129].

During the collision of two bodies, shock pressure will build up near the collision point [Fig. 11(a)]—collision point here refers to the point where a projectile and a target are first in contact or where the unshocked region of both the projectile and target is in contact at any instance during the impact process [130]. At the early stage of impact—jetless condition, the collision point moves ahead of the induced shock wave [Fig. 11(b)]. However, the speed of the collision point decreases with time and at a critical impact angle, α, the speed of the shock wave exceeds that of the collision point. This causes the pressure behind the shock to release at a free surface by the outward flow of fluid [131]—jetting condition [Fig. 11(c)]. The release or rarefaction wave interacts with the shock wave to develop tensile stresses within the sample [115, 132]. Jetting and material spalling therefore occur if the induced-tension exceeds the dynamic spall strength of the material [51, 133]; the scaling laws of jetting have since been found to be obeyed, both for spall strength and speed of sound [52]. Owing that the material has negligible strength upon the release of a shock wave, the motion in the collision region is considered a fluid hydrodynamics problem [130, 131]. Because the described jetting evolution is a natural dynamic effect involving pressure wave interactions, Hassani et al. [51, 52] concluded that jetting does not rely on ASI. Assadi et al. also observed some jetting at ${v}_{\text{i}}$ where no ASI occurred—simulated Cu impact at 500 m/s in their Fig. 5 [67]. As such, there should not be debate about whether or not jetting rely on ASI; clearly, it does not from both (Refs. [51, 67] observations.

##### ASI or jetting criteria for bonding

A fresh debatable question that spins from whether jetting requires ASI or not [51, 52, 65, 67, 127] is if both ASI and/or jetting can be taken as legitimate bonding criteria; i.e. can either of ${v}_{\text{ASI}}$ or ${v}_{\text{jetting}}$ be taken as ${v}_{\text{cr}}$. To seek evidence of post-mortem jetting at impact sites and its correlation to particle–substrate bonding, Tiamiyu e al. [25] conducted a detailed, systematic, and site-specific experimental study of single Cu microparticle impact on mirror-polished Cu substrate over a spectrum of ${v}_{\text{i}}$ as shown in Fig. 11(e). At lower ${v}_{\text{i}}$ (< 510 m/s), pure plastic indention with no upward flow of material at regions adjacent to the indent sites is observed. Meanwhile, both shear offsets and jetting indicated by white arrows can be seen on the substrate around the indentations as ${v}_{\text{i}}$ increases (510–588 m/s), yet without permanent particle adhesion. Although the type of indentation formed during impact depends on the materials involved [134], this observation shows that a change in indentation morphology is a function of *v*_*i*_ and could signal a change in indentation mechanism [135]. Shear offsets like those observed in Fig. 11(e) are signature of shear banding events in metals under compression, and by extension, they are imprints of ASI in impact events [136, 137].

The evolution of jetting together with these offsets at and between 510 and 588 m/s in Fig. 11(e) likely provides the first experimental evidence of both ASI and jetting occurring simultaneously prior to bonding, i.e. ${v}_{\mathrm{ASI}}\approx {v}_{\mathrm{jetting}}$, contrary to what modelling suggests [127]. In addition, the observation of bonding at ${v}_{\text{cr}}$—590 m/s and ~ 30 m/s higher in Fig. 11(e) without particle jetting importantly separates the onset of jetting from the onset of bonding. These observations suggest that both ASI criterion, ${v}_{\text{ASI}}={v}_{\text{cr}}$, in Ref. [45, 67] and jetting criterion, ${v}_{\mathrm{jetting}}={v}_{\text{cr}}$, are not valid for the onset of particle bonding, yet their onsets (ASI and jetting) may play a major role in producing clean contacting surfaces. This also agrees with the previous assertion that the onset of jetting does not necessarily produce metallurgical bonding [138].

##### What causes the departure of ${{\varvec{v}}}_{\mathbf{A}\mathbf{S}\mathbf{I}}$ and ${{\varvec{v}}}_{\mathbf{j}\mathbf{e}\mathbf{t}\mathbf{t}\mathbf{i}\mathbf{n}\mathbf{g}}$ from ${{\varvec{v}}}_{\mathbf{c}\mathbf{r}}$

Although ${v}_{\text{ASI}}$ and ${v}_{\text{jetting}}$ can be a good approximation of ${v}_{\text{cr}}$, there are possible reasons why ASI and jetting could occur prior to bonding, and by extension, the departure of ${v}_{\text{ASI}}$ and ${v}_{\text{jetting}}$ from ${v}_{\text{cr}}$. One of that is the extent of barriers to bonding present on either the particle and/or substrate as described in Sect. “Barriers to bonding: Extrinsic and Intrinsic”. Besides the possible effect of mismatch in the geometry of impacting bodies, more barriers (e.g. surface oxides, micropores, oxide dispersoids, and micron-sized SPP) on either particle or substrate sides can restrain local deformation and by extension limit the formation of jetting on either side [128]. Considering the jet evolution theory, even though a critical α value must be reached to transition from a jetless to a jetting condition in a single-particle impact, the wedge angles, ${\varphi }_{1}$—particle and ${\varphi }_{2}$—substrate, in Fig. 11(b) must also reach their critical values. During microparticle impact, α, ${\varphi }_{1}$, and ${\varphi }_{2}$ increases with time [129], and both particle and substrate jet at the same time only if ${\varphi }_{1}$ and ${\varphi }_{2}$ also reach their critical values at the same time. Hence, more barriers on one side, say particle, can result in an event where the shocked particle does not reach the critical condition for jetting simultaneously with the lesser barrier-substrate [129, 139], as schematized in Fig. 11(d) where only the substrate jets.

The surface roughness which is mostly overlooked in FEA models can also be an important contribution to the aforementioned departure. For instance, the modelling of a perfect well-rounded particle will transit from a jetless to an azimuthal jetting condition once a critical α is reached as schematized in Fig. 11(f). Whereas microparticles are usually not perfectly spherical, resulting in the azimuthal variation of α, and in turn, critical values of α are reached at different times during the impact process [Fig. 11(g)] [130]. This explains the evolution of discrete azimuthally separated jets that is not sufficient enough to cause permanent particle adhesion at and between 510 and 588 m/s in Fig. 11(e).

Therefore, we theorize that the condition where ASI and jetting criteria for bonding may likely hold is a case where *there are no barriers at all*, including geometry mismatch between the participating bodies in contact; this condition is hard to attain in cold spray and other related processes.

#### Melting

Melting is an interesting phenomenon that can set on below (rebound regime), within (bonding regime), and above (erosion regime) the deposition window—the range between ${v}_{\text{cr}}$ and erosion velocity, ${v}_{er}$. In this section, the focus is on the occurrence of melting in the rebound and bonding regime. For a very long time, findings support the notion that melting aids bonding. Although there are no microstructural details that validate metallurgical bonding occurs, Bae et al. [54] used FE-ALE method to show evidence of high-temperature thin molten layer at the softer side of Al/mild steel and Ti/Al interfaces; this results in lower ${v}_{\text{cr}}$ than in their matched Al/Al and Ti/Ti cases. Meanwhile, the liquid metal jetting formed by impact-induced melting was “suggested” to aid particle adhesion in an experimental cold spray process involving soft materials like Zinc [142]. Localized melting that “could” aid particle adhesion in harder Ti and Ti alloys has also been reported [55]; this is attributed to the poor thermal conductivity of the materials that in turn promote localized high interfacial temperature and ASI.

**Figure 12:**
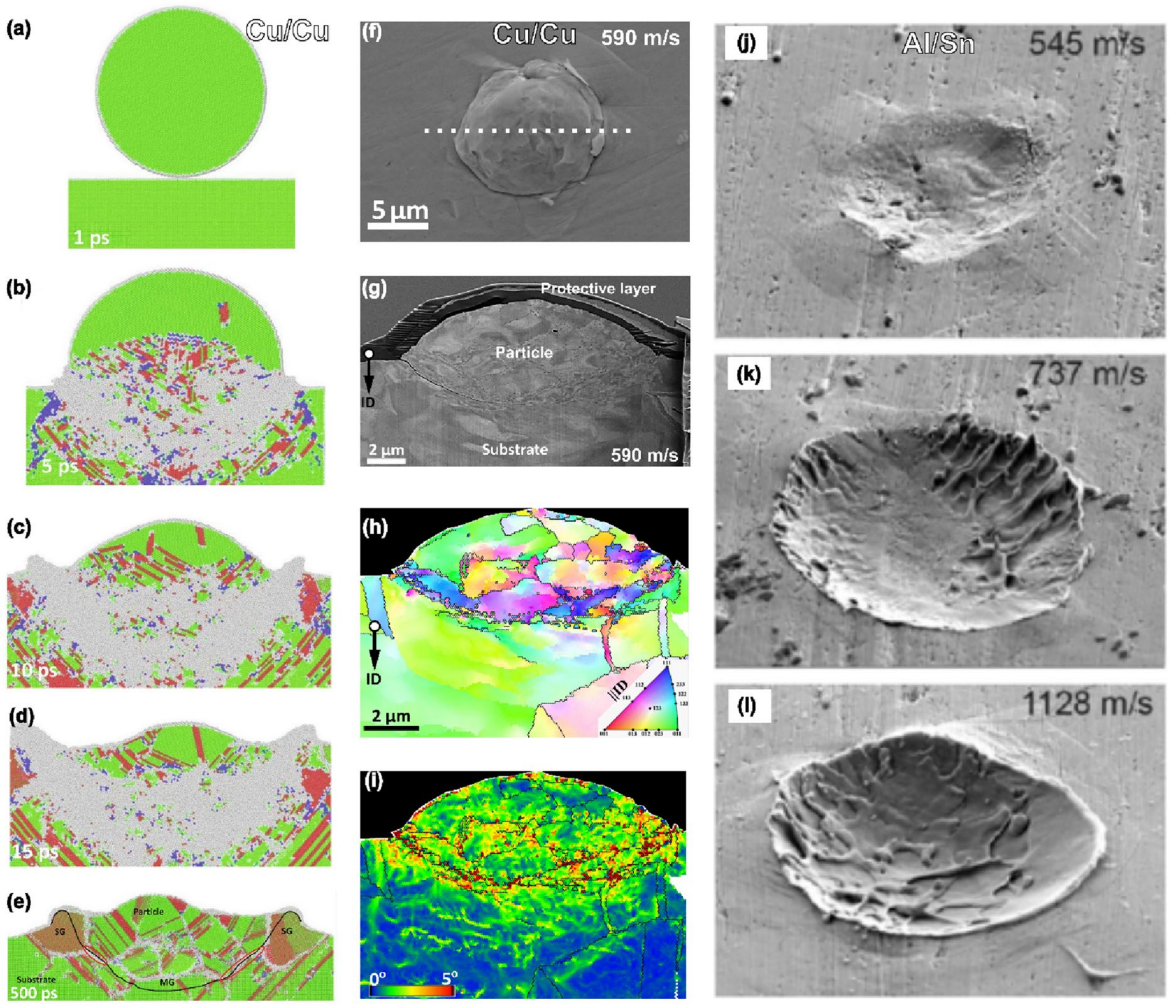
(a–e) Structural profile of copper particle/substrate system at different times, showing that melting may occur during impact, reprinted with permission from Ref. [56], copyright 2021 Elsevier; (f, g) SEM micrographs, (h) EBSD inverse pole figure map, (i) kernel average misorientation map showing no evidence of melting for adhered particle, launched at 590 m/s [140]; (j–l) SEM micrographs of Al microparticle impact-induced craters on a Zn substrate at different impact velocities, showing melting may occur but may not necessarily contribute to bonding, reprinted with permission from Ref. [141], copyright 2017 American Physical Society.

Using MD simulations for Cu/Cu impact pairs at 500 m/s [Fig. 12(a–e)], Reddy et al. [56] observed impact-induced local melting that promotes interfacial metallurgical bonding. Despite being similar particle/substrate material, their work [56] also suggests that mismatch in salient microstructural features like higher grain boundary area (amorphous phase) in the particle than the substrate slows heat dissipation rate to retain melting in the former than the latter. However, using LIPIT to launch Cu microparticle at 590 m/s, followed by post-mortem characterization of a lift-out lamella [140], Fig. 12(f–j) shows no evidence of melting. This is because the kernel average misorientation value—a measure of local misorientation and dislocation density—is higher (~ 5°) than what is typical for a recrystallized/annealed grain (below 1°) in Fig. 12(i).

Even when interfacial melting is observed to occur in a Cu/Stainless steel impact case [118], no sign of bonding is observed. In fact, Hassani et al. [141] experimentally observed for the first time that melting, if it occurs as shown in Fig. 12(j–l), can hinder bonding when the time for resolidification of melt is orders of magnitude longer than the particle residence time at the substrate. Except that melting hinders bonding as seen in Fig. 12(j–l), there is still no clear and direct evidence that melting aids bonding; this opens a new room for further studies.

#### Interface solid-state amorphization

Interfacial amorphization involves the formation of a compositionally gradient amorphous layer caused by atomic-scale intermixing/inter-atomic diffusion that accompanies severe plastic deformation [58]. This has been mainly observed in mismatch impact cases, such as Al-2Cu/4340 steel [57], Fe/Al [58], and Ni/Al-6061-T6 [143]. However, Kim et al. [144] reported a dynamic evolution of amorphous oxide layer that does not involve intermixing or inter-atomic diffusion in a single deposition of Ti particles on JIS S45C Steel, *although in a warm spraying process*. The authors (Kim et al.) posit that the dynamic evolution process was a result of the reoxidation of clean metal surface produced by prior ASI/jetting, and that the amorphous oxide produces strong bonded interfaces among the deposited titanium particles. Nevertheless, the process of amorphization, whether by intermixing, inter-atomic diffusion, or dynamic evolution of amorphous oxide layer during impact, has not gained enough support to play a role in bonding because the time in which particles and substrate are subject to high temperature and contact pressure is very short [92, 136].

*Subsection summary and perspective on bonding mechanisms* From the assessments in Sect. “Evolved phenomena during single microparticle impact and bonding mechanisms” above and the summary provided in Table 2, it is apparent that bonding in CS or any related processes is complex, and there is *no unified mechanism of bonding* due to the variability in barriers to bonding that are unique to each particle/substrate materials. There is rather only *a unified requirement for bonding*—clean metal–metal contact surfaces at sufficient ${v}_{\text{i}}$.

**TABLE 2:** Summarized previous works on single impact and their conclusions on contributions to bonding.

Method	Materials combination and references	Summarized contributions to bonding
LIPIT	Al/Al [104] Cu/Cu Ni/Ni Zn/Zn	Jet formation and subsequent material fragmentation are directly linked to bonding
	Al/Al [112]	Oxide layer composition, crystallinity, and thickness have significant influence on bonding
	Cu/Cu [114]	Amorphous carbon and spherical copper oxides are additional barriers to bonding, in addition to the well-known surface native oxides
	Cu/Cu [50]	Oxide layer delamination, in addition to oxide breakup contributes to clean metal–metal contact development. Jetting enhances the delamination process
Ballistic airgun	Pb/Pb [103]	Bonding is promoted where jetting and melting occurred at regions of maximum equivalent plastic strain and temperature
Wipe test	Ti/AlMg_3_ [23]	Bonding is dominantly caused by shear instabilities under plastic deformation and to a less extent by the heat of friction
	Cu/Al [101]	Mechanical adhesion is improved when both particle and substrate jet. High temperature and contact pressure at the interface improved bonding
	Ni/Stainless steel [145]	High contact pressure is the dominant factor for metallurgical bonding
FEM-CEL	Cu/Cu [51]	Jetting—a natural hydrodynamic effect that promotes bonding
Molecular dynamics	Cu/Cu [146]	Impact-induced melting promotes bonding
	Cu/Cu [147]	Surface oxide breakup to initiate metallurgical bonding
	Al/Al [91]	There is the formation of new grains at the particle-substrate interface due to grain boundary mobility and recrystallization which promote bonding

**Figure 13:**
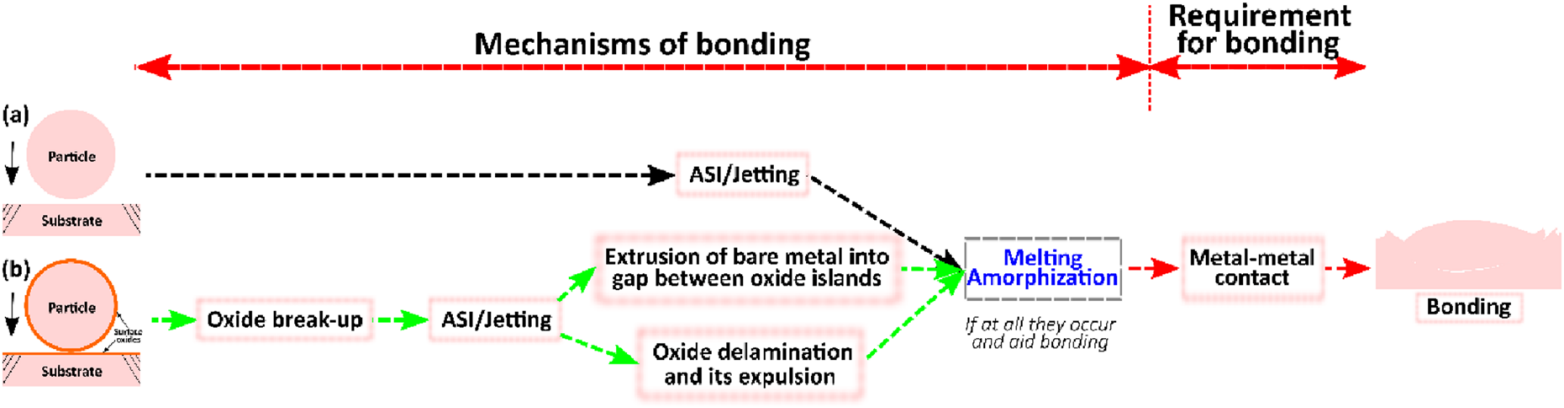
Combinations and possible sequence of evolving phenomena that inform the type of bonding mechanism for a sample case of (a) without and (b) with native surface oxides. In the instance where melting and/or amorphization does not occur and also aids bonding, it can be omitted from the sequence leading to bonding.

Therefore, each of the evolved phenomena in Sect. “Evolved phenomena during single microparticle impact and bonding mechanisms” cannot be firmly taken as a mechanism of bonding because multiple phenomena can coevolve. In other words, the existing barrier to bonding either extrinsic or intrinsic will affect the dominant bonding mechanism, and the energy “available” for bonding will first be used up to overcome these barriers to initiate metal–metal contact. For instance, in a pure metal, where no surface oxide or other barriers are present as schematized in Fig. 13(a), the excess dissipation energy beyond the plasticity of the particle impact is solely consumed by the jetting process to aid metal–metal contact and bonding at a much lower critical velocity than when the surface oxide layer is present. In the latter case [Fig. 13(b)], excess dissipation energy will first be used up to eliminate barriers, e.g. breaking and delamination of surface oxides, before metal–metal contact and bonding develops.

## Summary and outlook

A comprehensive review and assessments of the bonding mechanisms in single-particle impact events have been conducted. The evaluation of single-particle impact methods, barriers to bonding, and the reported physical phenomena/bonding mechanisms that set on are carefully presented. A major conclusion is that even though there is a unified condition in which bonding sets on—a pristine metal surface in contact at a sufficient impact velocity, there is no singular theory that can explain bonding mechanisms in CS. We posit that bonding mechanism is a function of the prevailing barriers unique to each impact scenario. As per the next steps for the CS community, we provide the following summary and outlook.

1. For bonding to set on in CS, the kinetic energy of the impinging particle must be sufficient to overcome the barriers (extrinsic and/or intrinsic) to bonding. This would provide the required pristine contact area for bonding to occur. Extrinsic barriers are well studied; hence, further studies on the role of dispersoids/second-phase particles to bonding are necessary to understand their effect on coating quality.
2. Barriers, mostly intrinsic, reinforce microstructure against plastic deformation and in turn jetting. Even in a similar impact case, like Cu/Cu, there are variability in the grain size and barriers on the particle and substrate sides, resulting in hardness mismatch. Further studies on the effect of barrier variabilities and hardness mismatch on the onset of jetting are required.
3. The most widely studied extrinsic barrier to bonding is surface oxides from both particles and substrates. As demonstrated in Sect. “Barriers to bonding: Extrinsic and Intrinsic”, these oxides significantly influence ${v}_{\text{cr}}$. It is therefore important that powder particle production and handling for CS process should be optimized to reduce the risk of excessive oxide contaminations that are difficult to eliminate. Following the acid pickling process by Li et al. [148], single microparticle testing of near-oxide-free particle will be desirable. This will also allow the unambiguous evaluation of oxide thickness role on ${E}_{\text{d}}^{\text{max}}$, DVR, and by extension ${v}_{\text{cr}}$.
4. Both ASI and jetting contribute to bonding in CS as they significantly promote the development of a clean metal surface required for adhesion. Highlighted barriers and geometry of contacting bodies determine the initiation of these phenomena. Based on Sect. “Adiabatic shear instability and jetting”, ASI is still indirectly deduced from the evolution of shear offsets; more experimental work is needed to ascertain their onsets.
5. There is clear evidence that melting can hinder bonding. However, that melting aid bonding is still a subject of debate; more clear and direct experimental evidence on the role of melting on bonding is required.

## Data Availability

The datasets generated during and/or analysed during the current study are available from the corresponding author on reasonable request.
